# Supplementary material for: Supervised deep learning with gene functional annotation for cell classification
Source: PLoS Comput Biol. 2026 Jun 1;22(6):e1014327. doi: 10.1371/journal.pcbi.1014327 (PMC13235940; doi:10.1371/journal.pcbi.1014327)
Supplement: S1 Appendix — (PDF) [file pcbi.1014327.s001.pdf]

Supplementary materials for "Supervised deep learning with gene annotation for cell classification"

Zhexiao Lin
Department of Statistics, University of California, Berkeley, CA, USA

Yuanyuan Gao
Department of Statistics, University of California, Berkeley, CA, USA

Wei Sun
Public Health Sciences Division, Fred Hutchinson Cancer Center, WA, USA
Department of Biostatistics, University of Washington, Seattle, WA, USA
Department of Biostatistics, University of North Carolina, Chapel Hill, NC, USA

May 24, 2026

A Prediction of COVID-19 severity

Su et al. (Su et al., 2020) collected scRNA-seq data from approximately 0.55 million peripheral blood mononuclear cells (PBMCs) from 254 COVID-19 samples at two time points: baseline (BL; initial clinical diagnosis) and AC (a few days after the initial clinical diagnosis). Our objective was to predict COVID-19 severity using the 129 baseline samples. We identified CD4+ T cells and CD8+ T cells using paired single-cell TCR data. The gene expression data, downloaded from ArrayExpress, were already corrected for read depth and log-transformed. We converted the expression of gene j in cell i, denoted by x_{ij}, to count data using the transformation $y_{ij} = (\exp(x_{ij}) - 1)/d_i$, where $d_i = \exp(\min_j(x_{ij})) - 1$ is the inferred read depth for cell i. After removing a small proportion of cells with relatively high mitochondrial gene expression, 73,383 CD4+ T cells and 40,223 CD8+ T cells remained. Both the CD4+ T-cell and CD8+ T-cell datasets initially contained 24,966 genes. We then normalized each cell so that the total count equaled 10,000 and log-transformed the data. We excluded all mitochondrial genes from downstream analyses and retained only genes expressed in more than 2% of cells. This resulted in 7,704 genes for CD4+ T cells and 7,864 genes for CD8+ T cells.

To define disease severity labels, we used the World Health Organization (WHO) ordinal scale: WHO scores 1–2 were classified as mild, 3–4 as moderate, and 5–7 as severe. Each

cell was assigned the label of the corresponding patient. For the present analysis, we included only data from 49 patients with mild disease and 32 patients with severe disease. Among these, there were 25,687 and 16,334 CD4+ T cells from patients with mild and severe disease, respectively, and 16,449 and 7,671 CD8+ T cells from patients with mild and severe disease, respectively. We randomly split the patients in the mild and severe groups into two approximately equal subsets, using one subset for training and the other for testing. For this dataset, as well as for all other datasets analyzed in this study, we randomly designated 10% of the cells in the training data as validation data and used the remaining cells for model fitting.

For CD8+ T cells, we identified 649 differentially expressed genes (FDR ≤ 0.05) with higher expression in mild cases and 2,926 differentially expressed genes with higher expression in severe cases. By default, we retained at most 1,000 marker genes with the highest normalized variance for each class, resulting in 1,649 genes in total (649 for mild and 1,000 for severe). We varied the weight parameter over the set $\{0, 0.25, 0.5, 1, 2, 5, 10\}$. The corresponding cell-level AUC values were $\{0.898, 0.890, 0.879, 0.884, 0.878, 0.857, 0.851\}$, and the individual-level AUC values were $\{0.967, 0.970, 0.976, 0.958, 0.964, 0.973, 0.955\}$.

We used the `goseq` function in R (Young et al., 2010) to assess whether each gene set identified by SDAN was enriched for genes from known functional categories. We considered three classes of functional categories obtained from MSigDB (Liberzon et al., 2015):

- Gene Ontology biological process terms: `c5.go.bp.v2023.2.Hs.symbols.gmt`.
- Reactome pathways: `c2.cp.reactome.v2023.2.Hs.symbols.gmt`.
- Immune-related categories: `c7.all.v2023.2.Hs.symbols.gmt`.

Multiple testing across functional categories was controlled using the Benjamini–Hochberg FDR procedure.

The number of enriched functional categories increased as the weight on the unsupervised loss increased from 0.25 to 2 (Supplementary Fig A). No functional category was enriched when the weight was 0. This pattern is expected because a larger weight on the unsupervised loss encourages the identification of gene sets with more annotated gene–gene interactions within each set.

For CD4+ T cells, we identified 1,126 differentially expressed genes for mild cases and 2,057 for severe cases. From these, we selected 2,000 genes, with 1,000 genes from each class. We again varied the weight parameter over the set $\{0, 0.25, 0.5, 1, 2, 5, 10\}$. The resulting cell-level AUC values were $\{0.907, 0.914, 0.909, 0.906, 0.897, 0.884, 0.829\}$, and the individual-level AUC values were $\{0.940, 0.940, 0.952, 0.958, 0.949, 0.952, 0.955\}$.

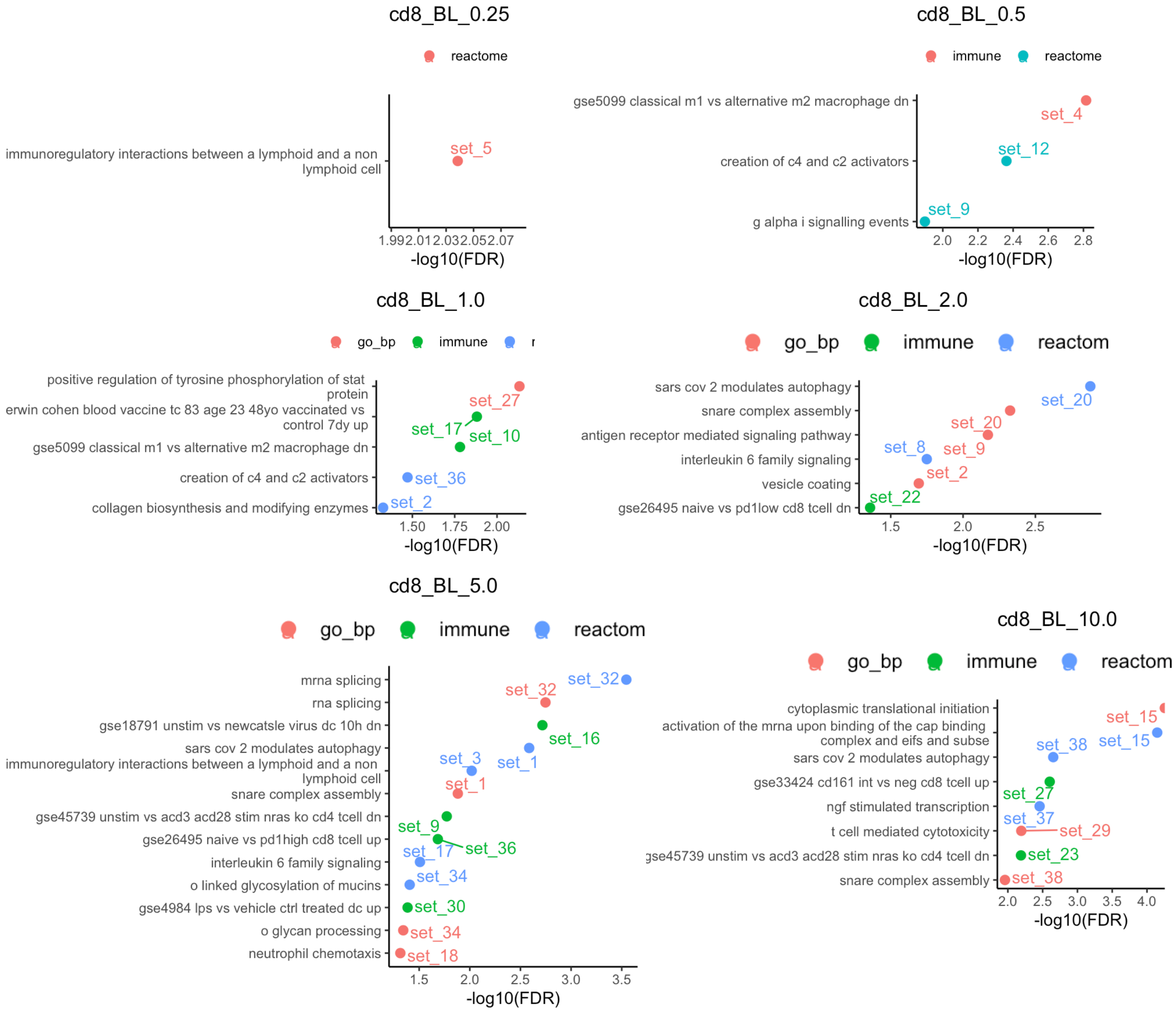

Figure A: Functional category enrichment results for gene sets identified from CD8+ T cells under different weights assigned to the unsupervised loss.

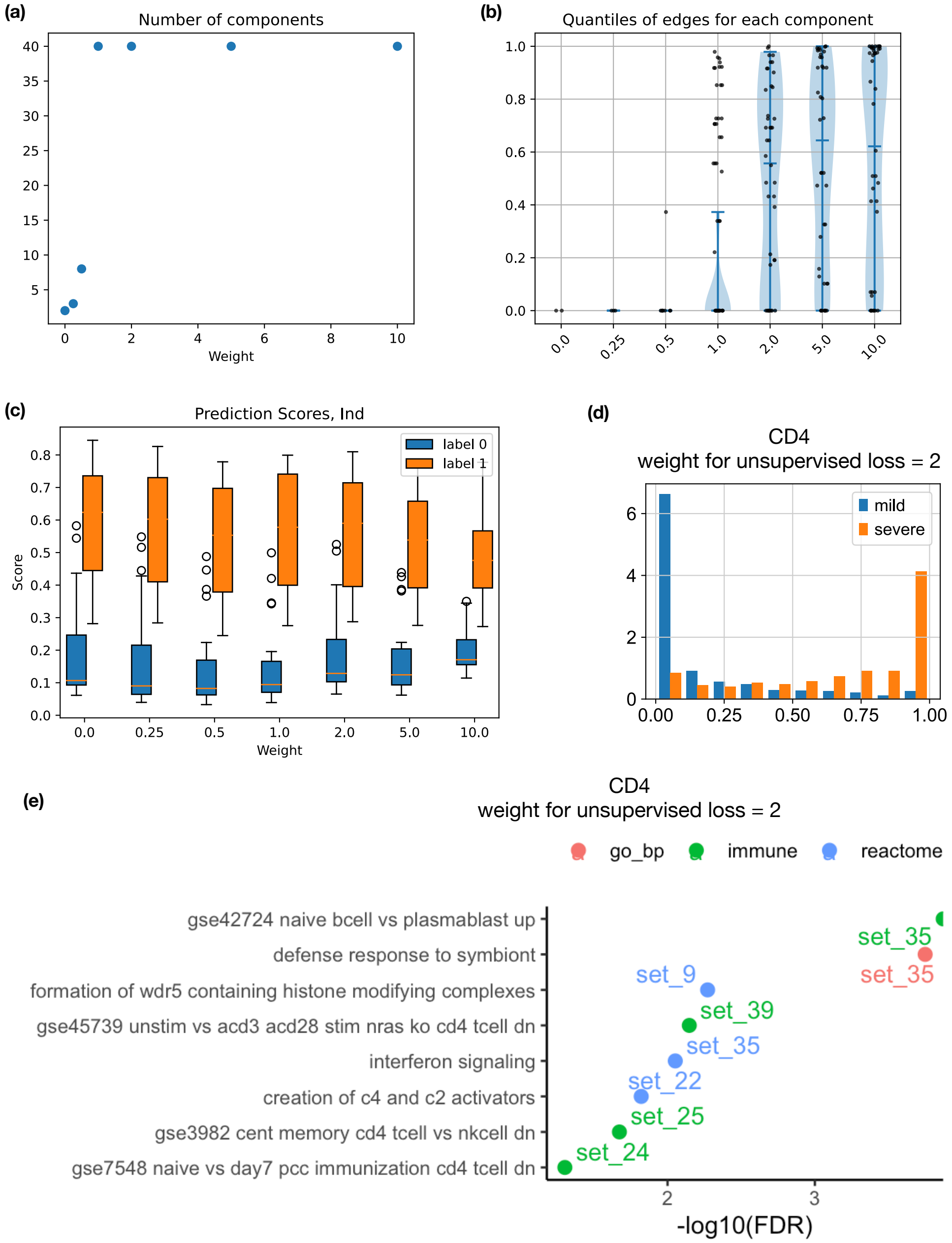

Figure B: Summary of results for CD4+ T cells. **(a)** Number of latent gene programs under different weights for the unsupervised loss. **(b)** Violin plots of connection quantiles. Each point represents one gene program, and the connection quantile is computed relative to the number of connections observed in randomly selected gene sets of the same size. **(c)** Box plots of individual-level severe-disease scores, defined as the average of cell-level prediction scores, across different weights for the unsupervised loss. **(d)** Distribution of cell-level prediction scores for severe disease in the testing data, stratified by the severe versus mild disease status of the corresponding individual. **(e)** Enriched functional categories of the identified gene sets when the weight for the unsupervised loss is 2.

B Prediction of dementia

We used single-nucleus RNA-seq (snRNA-seq) data from the Seattle Alzheimer's Disease Cell Atlas (SEA-AD) consortium (Gabitto et al., 2024). This dataset consisted of multi-omic measurements from the middle temporal gyrus (MTG) of 84 donors. The MTG is a brain region relevant to dementia because it is involved in language, semantic memory, and higher-order visual processing. We analyzed gene expression in two cell types: astrocytes (Astro) and microglia/perivascular macrophages (Micro-PVM). There were 70,009 Astro cells and approximately 40,000 Micro-PVM cells. Both the Astro and Micro-PVM datasets initially contained 36,517 genes. We normalized the gene expression of each cell so that the total UMI count was 10,000 and then log-transformed the data. We excluded all mitochondrial genes and retained only genes expressed in more than 2% of cells. This resulted in 16,058 genes for Astro and 13,895 genes for Micro-PVM. Each cell was labeled according to the cognitive status of its donor. There were 42 donors with dementia and 42 donors without dementia. We randomly selected half of the donors in each group to construct the training data and used the remaining donors as the testing data.

For Astro cells, we identified 6,765 differentially expressed genes for the non-dementia class and 2,120 for the dementia class. From these, we selected 2,000 genes. We varied the weight parameter over the set $\{0, 0.25, 0.5, 1, 2, 5, 10\}$ and obtained the following cell-level AUC values: $\{0.740, 0.726, 0.726, 0.722, 0.715, 0.677, 0.667\}$. The corresponding individual-level AUC values were $\{0.748, 0.739, 0.748, 0.751, 0.744, 0.744, 0.746\}$ (Supplementary Fig C).

For Micro-PVM cells, we identified 2,554 differentially expressed genes for the non-dementia class and 1,939 for the dementia class. From these, we selected 2,000 genes. Varying the weight parameter over the same set of values, the cell-level AUC values were $\{0.648, 0.650, 0.643, 0.650, 0.634, 0.620, 0.602\}$, and the individual-level AUC values were $\{0.719, 0.714, 0.726, 0.737, 0.735, 0.728, 0.730\}$ (Supplementary Fig D).

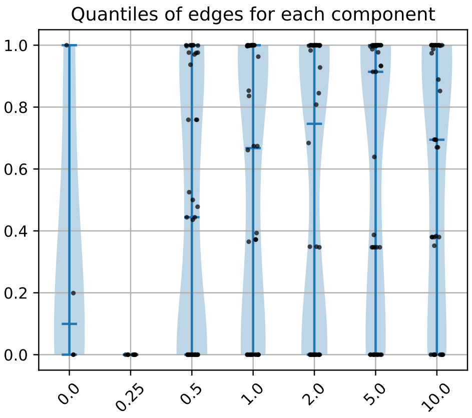

(a)

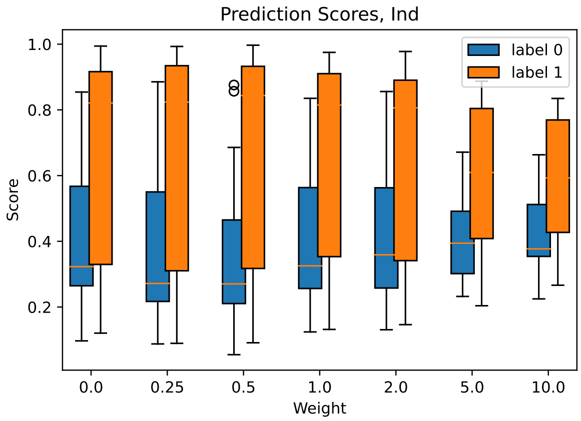

(b)

Figure C: Evaluation of different weights for the unsupervised loss using snRNA-seq data from **astrocytes**. **(a)** Violin plots of connection quantiles. Each point represents one gene program. The connection quantile (y-axis) is computed relative to the number of connections observed in randomly selected gene sets of the same size. **(b)** Box plots of individual-level SDAN prediction scores, defined as the average of cell-level prediction scores, across different weights for the unsupervised loss.

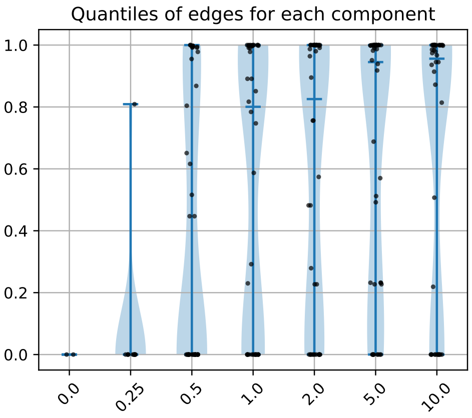

(a)

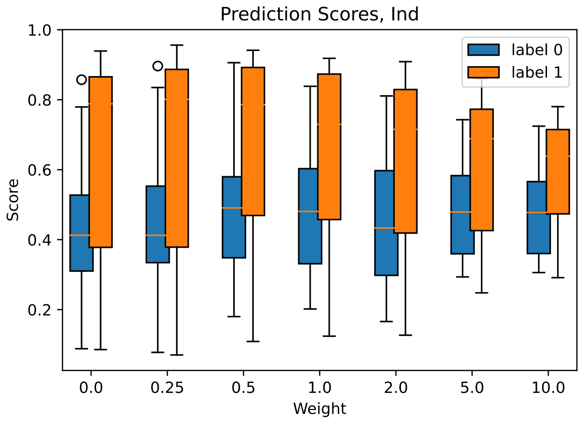

(b)

Figure D: Evaluation of different weights for the unsupervised loss using snRNA-seq data from **Micro-PVM**. **(a)** Violin plots of connection quantiles. **(b)** Box plots of individual-level SDAN prediction scores.

C Prediction of patient response to cancer immunotherapy

Because existing datasets have limited sample sizes, we combined two datasets to study response to cancer immunotherapy. The training data were obtained from Sade-Feldman et al. (2018) and consisted of 6,350 CD8+ T cells and 36,602 genes from 17 responding tumors and 31 non-responding tumors. The data had already been normalized, and we therefore used them directly. We excluded all mitochondrial genes and retained only genes expressed in more than 2% of cells, leaving 13,480 genes. Each cell was labeled according to the corresponding patient's response to immunotherapy. All data from Sade-Feldman et al. (2018) were used for training. We evaluated predictive performance using the dataset from Yost et al. (2019), which consisted of 27,924 CD8+ T cells and 18,189 genes from 15 patients, including 8 responders and 7 non-responders.

We identified 7,112 differentially expressed genes for the non-response group and 259 for the response group. From these, we selected 1,259 genes. We varied the weight parameter over the set $\{0, 0.25, 0.5, 1, 2, 5, 10\}$ and obtained the following cell-level AUC values: $\{0.572, 0.587, 0.590, 0.518, 0.532, 0.531, 0.553\}$. The corresponding individual-level AUC values were $\{0.732, 0.821, 0.804, 0.643, 0.661, 0.482, 0.607\}$ (Supplementary Fig E).

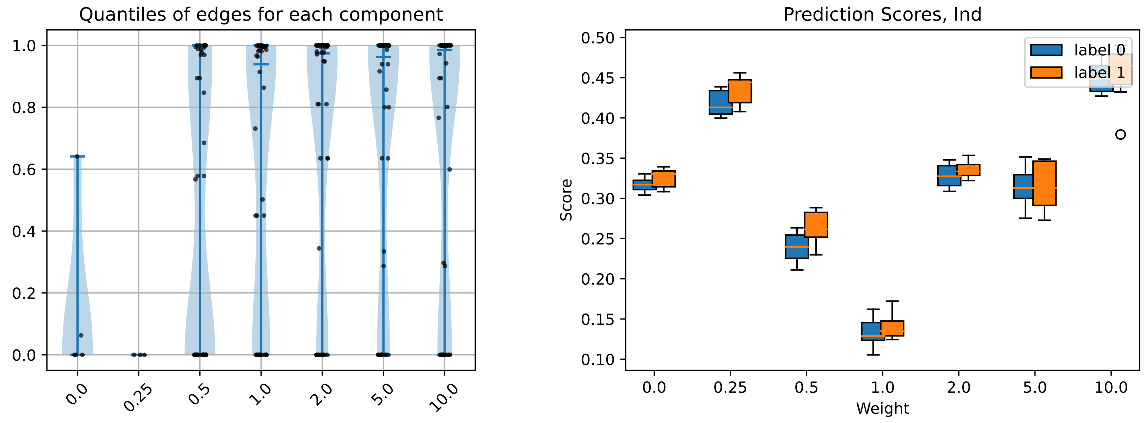

(a) (b)

Figure E: Evaluation of different weights for the unsupervised loss using scRNA-seq data from CD8+ T cells to predict cancer immunotherapy response. **(a)** Violin plots of connection quantiles. **(b)** Box plots of individual-level SDAN prediction scores.

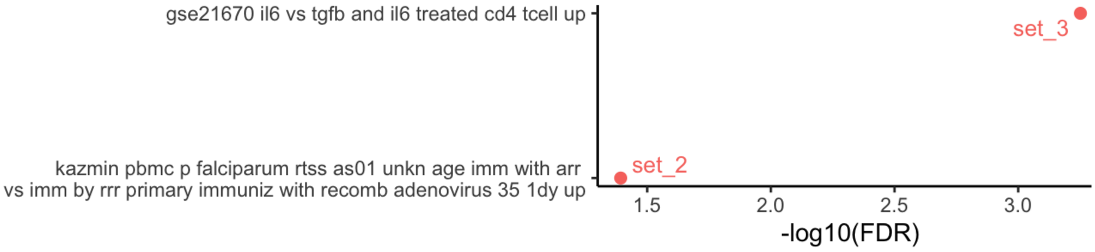

(a) Weight 0.25 for the unsupervised loss.

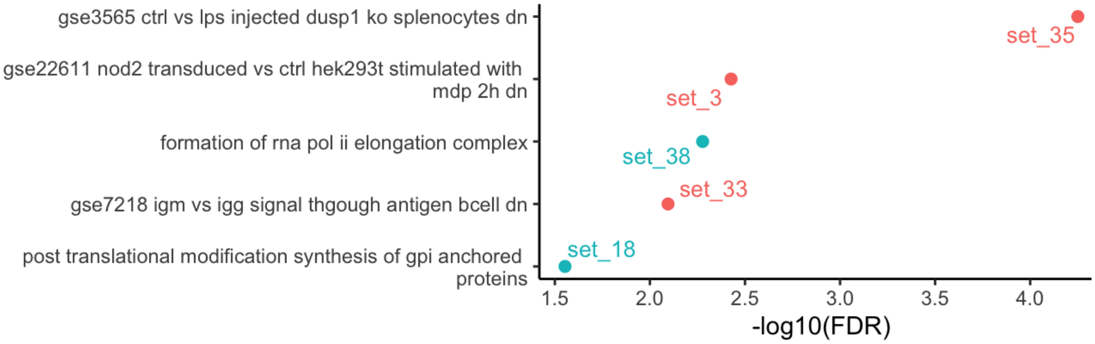

(b) Weight 0.5 for the unsupervised loss.

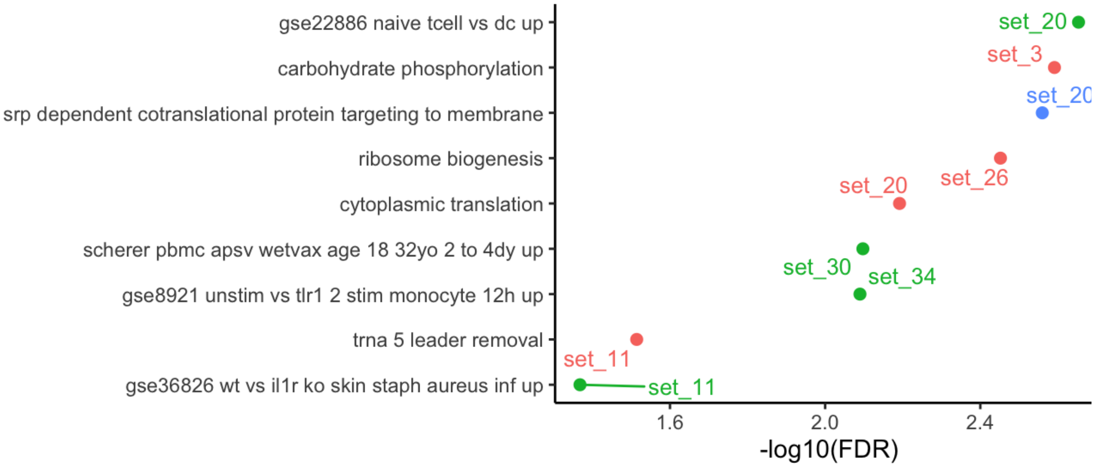

(c) Weight 1.0 for the unsupervised loss.

Figure F: Functional category enrichment analysis across different values of the unsupervised-loss weight using scRNA-seq data from CD8+ T cells to predict cancer immunotherapy response.

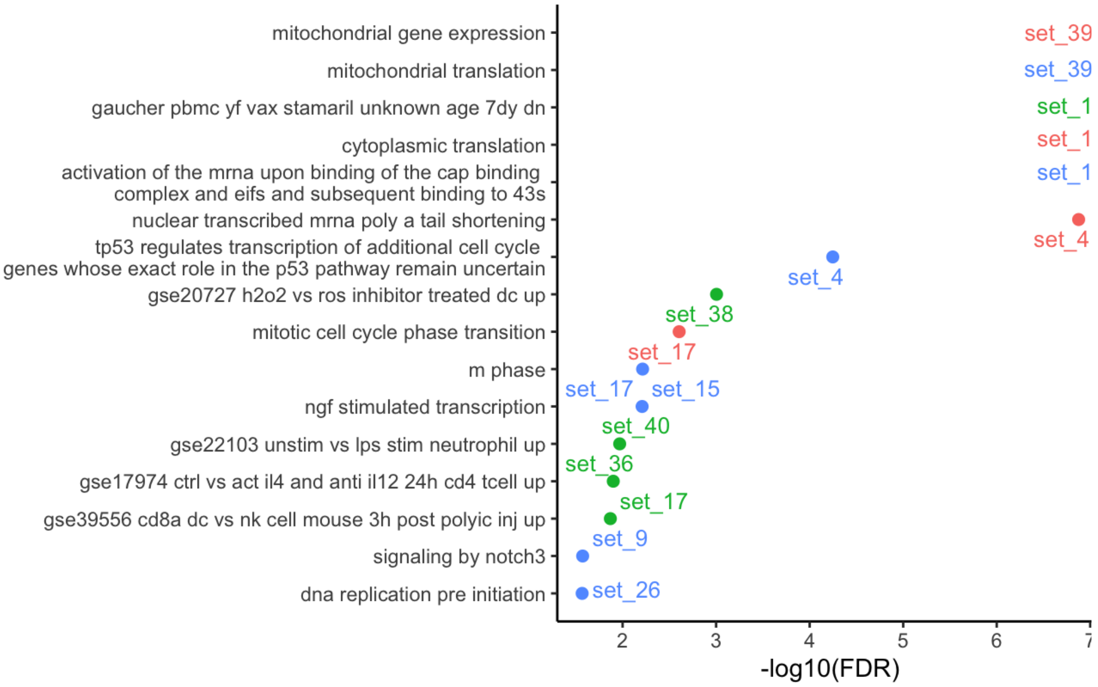

(a) Weight 5.0 for the unsupervised loss.

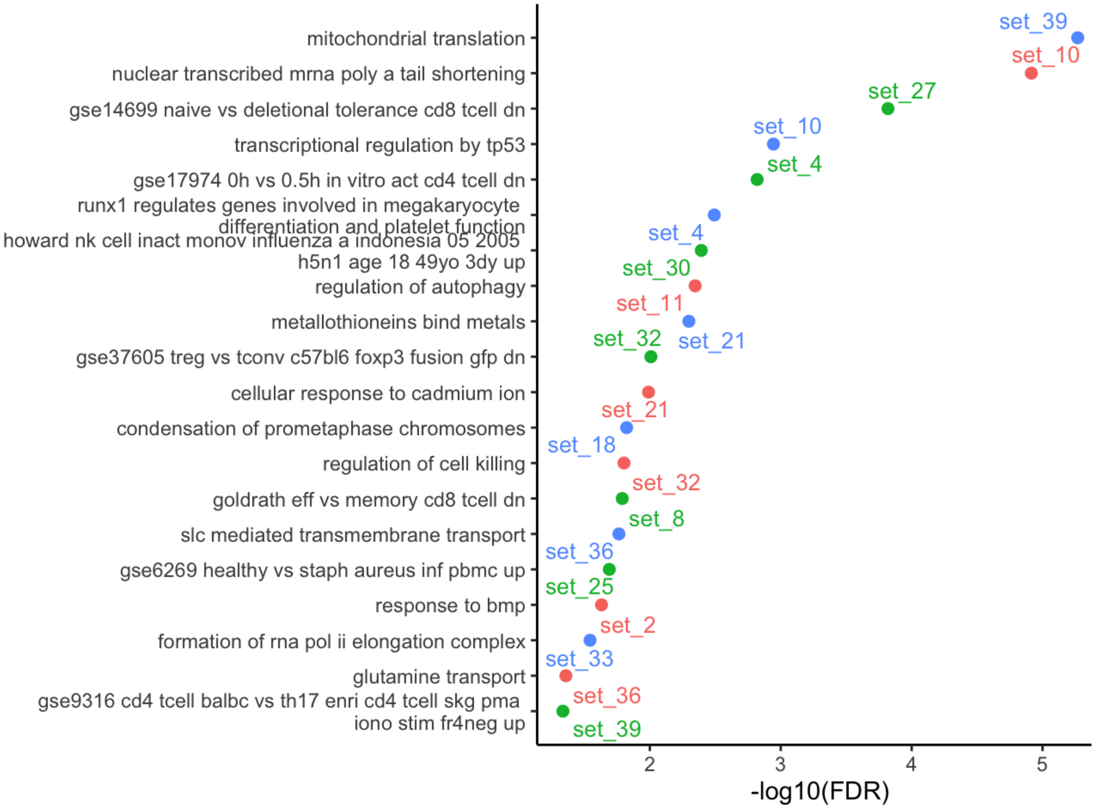

(b) Weight 10.0 for the unsupervised loss.

Figure G: Continuation of the functional category enrichment analysis across different values of the unsupervised-loss weight using scRNA-seq data from CD8+ T cells to predict cancer immunotherapy response.

Table A: Gene set 1 identified by SDAN.

hgnc_symbol	chr	description
ATPAF1	chr1	ATP synthase mitochondrial F1 complex assembly factor 1
CDK5RAP1	chr20	CDK5 regulatory subunit associated protein 1
DHX58	chr17	DExH-box helicase 58
EARS2	chr16	glutamyl-tRNA synthetase 2, mitochondrial
GTPBP10	chr7	GTP binding protein 10
KHSRP	chr19	KH-type splicing regulatory protein
MALSU1	chr7	mitochondrial assembly of ribosomal large subunit 1
MCUB	chr4	mitochondrial calcium uniporter dominant negative subunit beta
MRPL23	chr11	mitochondrial ribosomal protein L23
MRPL48	chr11	mitochondrial ribosomal protein L48
MRPL55	chr1	mitochondrial ribosomal protein L55
MRPS33	chr7	mitochondrial ribosomal protein S33
MRRF	chr9	mitochondrial ribosome recycling factor
MTFMT	chr15	mitochondrial methionyl-tRNA formyltransferase
MTIF2	chr2	mitochondrial translational initiation factor 2
MTO1	chr6	mitochondrial tRNA translation optimization 1
MTRES1	chr6	mitochondrial transcription rescue factor 1
NDUFAF4	chr6	NADH:ubiquinone oxidoreductase complex assembly factor 4
NDUFAF5	chr20	NADH:ubiquinone oxidoreductase complex assembly factor 5
NDUFS4	chr5	NADH:ubiquinone oxidoreductase subunit S4
PHAX	chr5	phosphorylated adaptor for RNA export
SARS2	chr19	seryl-tRNA synthetase 2, mitochondrial
ZNF431	chr19	zinc finger protein 431

D Sensitivity analysis

We conducted sensitivity analyses using the peripheral blood mononuclear cell (PBMC) single-cell RNA-seq dataset (Zheng et al., 2017), which serves as the primary benchmark dataset in this study. This dataset consists of purified immune cell populations profiled using the 10x Genomics platform. In our processed analysis, we retained 9 cell types after excluding CD34+ cells because of their comparatively low purity. For the sensitivity analysis, we focused on the binary classification task of distinguishing `cd4_t_helper` cells from `naive_t` cells. The dataset contains 11,177 `cd4_t_helper` cells and 10,460 `naive_t` cells. This comparison provides a stringent evaluation setting because these two populations are closely related in lineage while remaining transcriptionally distinguishable. The PBMC dataset therefore provides an appropriate and biologically meaningful framework for assessing the robustness of SDAN. In this section, we examine the sensitivity to the gene annotation and to DE-based gene preselection.

D.1 Sensitivity to gene annotation

To evaluate the sensitivity of SDAN to the gene annotation graph, we performed a controlled graph-perturbation analysis. We used the same preprocessing pipeline as in the main SDAN analysis and selected DE genes using the standard procedure, retaining the top 1,000 genes so that only the annotation graph, rather than the gene set itself, varied across sensitivity settings.

We constructed the baseline gene annotation graph by mapping the selected genes onto BioGRID protein–protein interactions. We then perturbed this graph by replacing a specified fraction of undirected edges with randomly sampled new edges while preserving the total number of edges. The perturbation fraction ranged from 0 to 1, where 0 corresponds to the original annotation graph and 1 corresponds to a fully rewired graph with the same number of edges. For each perturbation setting, SDAN was retrained using identical model hyperparameters and the same graph-regularization weight. We then carried out post hoc analyses of predictive performance and of the learned loading matrix S across perturbation settings.

Supplementary Table B summarizes the loss decomposition and predictive performance. The results indicate that as the annotation graph becomes increasingly perturbed, SDAN largely preserves classification performance but changes how genes are represented. Specifically, the test AUC remains fairly stable across perturbation levels, indicating that predictive signal is retained, whereas the loss decomposition shifts markedly: $\mathcal{L}_{\mathrm{o}}$ becomes much larger and $\mathcal{L}_{\mathrm{c}}$ remains close to its most favorable value. This pattern suggests that, under a corrupted graph, the model no longer learns sparse, well-separated gene programs like those obtained from the original graph. Instead, it appears to rely on more diffuse and overlapping loading patterns that still support classification and can perform well on the minCUT term, but no longer satisfy the desired orthogonality structure. This interpretation is consistent with the sharp drop in Hoyer sparsity shown in Supplementary Fig H, which suggests that the loading matrix becomes flatter and less structured after perturbation. The model does not simply converge to a random partition of genes into gene programs of similar

size, because SDAN minimizes the full objective, $\mathcal{L}_{\text{clf}} + w(\mathcal{L}_c + \mathcal{L}_o)$, rather than the graph regularization terms alone. A random balanced partition would generally fail to preserve phenotype-relevant signal and therefore would not be favored by the classification term. In addition, the loading matrix is not optimized as an unconstrained partition; rather, it is generated through the graph-convolution and softmax parameterization. As a result, after graph perturbation, optimization may be driven toward a different local solution characterized by overlapping gene programs rather than a clean random partition. Taken together, these results suggest that a biologically meaningful annotation graph helps SDAN learn interpretable and well-separated gene programs, whereas a perturbed graph causes the model to sacrifice orthogonality and interpretability in order to maintain predictive accuracy.

Table B: Post hoc loss decomposition across annotation-graph perturbation settings for the `cd4_t_helper` vs. `naive_t` classification task.

Perturbation	$\mathcal{L}_{\text{c}}$	$\mathcal{L}_{\text{o}}$	$\mathcal{L}_{\text{clf}}$	Test AUC
0.0	-0.7572	0.3065	0.3575	0.8650
0.1	-0.9568	1.1986	0.3653	0.8621
0.2	-0.9693	1.2555	0.4070	0.8674
0.3	-0.9820	1.2865	0.4463	0.8659
0.4	-0.9814	1.2946	0.3866	0.8784
0.5	-0.9794	1.2942	0.4036	0.8854
0.6	-0.9803	1.2946	0.4579	0.8594
0.7	-0.9814	1.2949	0.4190	0.8747
0.8	-0.9832	1.2953	0.4193	0.8722
0.9	-0.9820	1.2950	0.6695	0.8598
1.0	-0.9825	1.2953	0.3788	0.8822

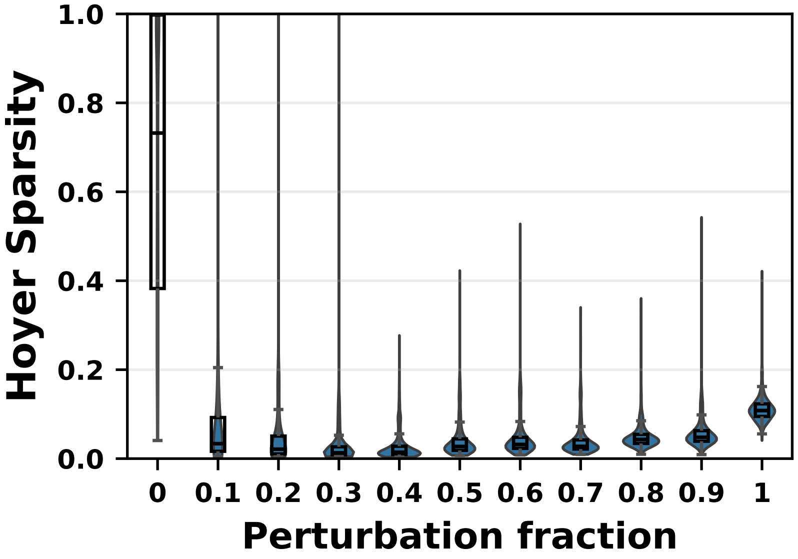

Figure H: Hoyer sparsity of the learned loading matrix across annotation-graph perturbation settings.

D.2 Sensitivity to DE-based gene preselection

To assess sensitivity to DE-based gene preselection, we repeated the SDAN analysis while varying the false discovery rate (FDR) threshold used to define differentially expressed genes. We used the same preprocessing pipeline as in the main SDAN analysis. For each FDR setting, DE analysis was performed separately for `cd4_t_helper` and `naive_t` cells, and the resulting p-values were adjusted using the Benjamini–Hochberg procedure. We then defined the DE gene set as the uncapped union of all genes with adjusted p-value less than or equal to the chosen FDR threshold across the two cell types. We considered the thresholds $\{0.05, 0.1, 0.2, 0.3, 0.4, 0.5\}$.

For each selected DE gene set, we reconstructed the corresponding gene annotation graph using the same BioGRID-based annotation framework and retrained SDAN with identical model settings. This design isolates the effect of DE preselection while keeping the downstream architecture, optimization procedure, and evaluation protocol unchanged. Model performance was evaluated on the test data using the test AUC, allowing us to assess whether SDAN remained stable as the stringency of DE filtering changed and as the number of retained genes varied. Beyond predictive performance, we also evaluated the stability of the learned gene programs across FDR settings. For each trained model, we used the selected DE genes and the learned loading matrix S to construct the final gene sets corresponding to the gene programs using the same thresholding rule applied in the downstream interpretation step. We then compared these gene sets across FDR values using two similarity metrics: the Jaccard index and the adjusted Rand index (ARI).

Supplementary Table C shows that the number of DE genes increases monotonically as the FDR threshold is relaxed, from 1,858 genes at FDR 0.05 to 4,369 genes at FDR 0.50. In contrast, the number of final selected genes derived from the learned loading matrix remains much smaller and relatively stable, staying in the range of approximately 200–270 genes. This indicates that increasing the number of DE genes does not lead to a proportional increase in genes whose loadings exceed the selection threshold. At the same time, the proportion of empty gene programs rises from 0.65 to 0.90, indicating that the additional DE genes admitted at less stringent thresholds are generally not converted into additional active gene programs. Together, these results suggest that the model concentrates signal into a limited subset of genes and a relatively small number of active gene programs, whereas many of the additional DE candidates introduced at higher FDR thresholds receive low weights and do not survive the SDAN selection step.

Supplementary Fig I shows that the selected gene sets remain moderately to strongly similar across FDR settings according to the Jaccard index, especially for neighboring thresholds. The ARI-based similarity values further indicate that shared genes are usually assigned to similar gene programs across runs. Taken together, these results suggest that DE analysis in SDAN primarily serves as a broad screening step, whereas the model itself concentrates signal into a stable core set of genes and gene-program assignments. In other words, the biologically interpretable representation learned by SDAN is driven more by the downstream supervised, graph-regularized learning step than by the exact upstream DE cutoff.

Table C: Number of DE genes, number of final selected genes, average number of final selected genes among non-empty gene programs, and proportion of empty gene programs across FDR settings.

FDR	# DE genes	# final genes	average # of final genes	Prop. of empty
0.05	1858	234	16.7143	0.6500
0.10	2243	203	25.3750	0.8000
0.20	2855	229	28.6250	0.8000
0.30	3411	271	38.7143	0.8250
0.40	3793	240	48.0000	0.8750
0.50	4369	236	59.0000	0.9000

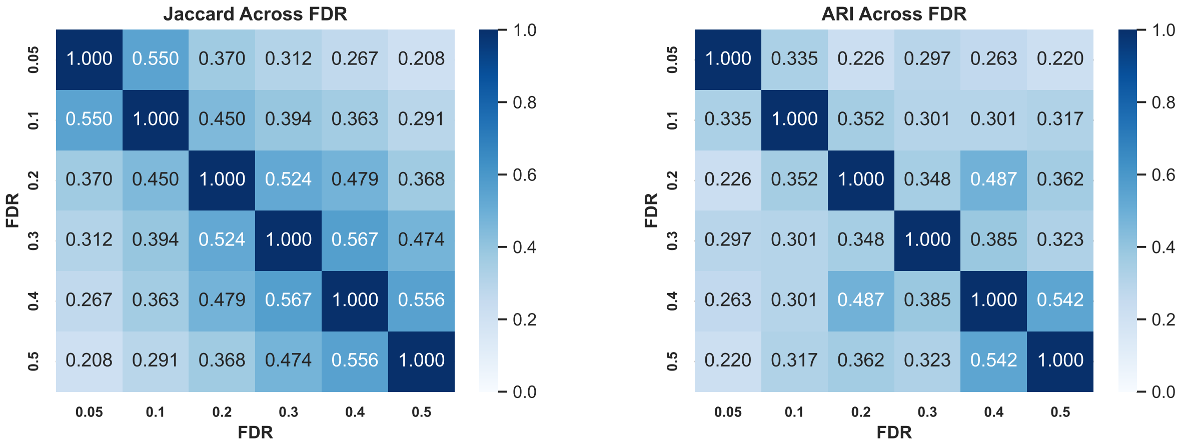

(a) (b)

Figure I: Comparison of similarity metrics across DE FDR settings. **(a)** Jaccard index of the final selected genes across DE FDR settings. **(b)** ARI of gene-program assignments based on overlapping genes across DE FDR settings.

E Comparison with DE + Enrichment

We compared SDAN with a classical differential-expression-plus-enrichment (DE + enrichment) pipeline using the same datasets analyzed throughout this study.

To construct the classical baseline, we first identified DE genes using the same procedure as in SDAN. We then performed functional enrichment analysis using three curated annotation collections: Gene Ontology Biological Process, Reactome, and immune-related gene sets. For each annotation term, enrichment among the DE genes was assessed using a hypergeometric test, with the gene universe defined as the set of genes present in the dataset. Multiple testing was controlled using the Benjamini–Hochberg procedure, and terms with FDR ≤ 0.05 were considered significantly enriched. Because classical enrichment analysis often yields many highly overlapping terms, we further reduced redundancy by applying sequential Jaccard filtering. Specifically, enriched terms were first ranked by adjusted significance, and each term was retained only if its gene set was sufficiently distinct from those already selected. We used a Jaccard similarity threshold of 0.5. The top 40

nonredundant enriched terms were then retained to form the final classical pathway panel, matching the default number of gene programs used by SDAN.

We first compared the overlap between SDAN programs and any of the enriched terms (i.e., functional categories) identified by the "DE + Enrichment" baseline method. Among the 200 SDAN gene programs, only 46 have significant overlaps with the baseline terms (Supplementary Table D, Supplementary Fig J). If we focus on 32 "informative" SDAN programs that have high AUCs to classify individuals of two classes (AUC > 0.6) and high connectivity quantile (> 0.7), only 8 of them have significant overlap with baseline terms (Supplementary Table D).

Table D: Counts of SDAN programs whose best-matching baseline term overlap is significant at the indicated BH-adjusted p-value thresholds, both among programs passing the filter (individual level AUC > 0.6 and connectivity quantile > 0.7) and across all 40 SDAN programs per cell type.

	Filtered			All		
Sheet	N	$p_{\text{adj}} < 0.01$	$p_{\text{adj}} < 10^{-4}$	N	$p_{\text{adj}} < 0.01$	$p_{\text{adj}} < 10^{-4}$
SEA-AD Astro	8	3	0	40	6	1
SEA-AD Micro-PVM	10	1	0	40	7	0
Su et al. (2020) $CD4^{+}$ T	4	2	0	40	15	1
Su et al. (2020) $CD8^{+}$ T	6	1	0	40	13	2
Yost et al. (2019) $CD8^{+}$ T	4	1	1	40	5	3
**Total**	**32**	**8**	**1**	**200**	**46**	**7**

Next we assess whether these baseline enriched terms can classify outcomes. To convert these enriched terms into predictive features, we computed a per-cell pathway activity score for each term, defined as the average expression of the genes in that term after subtracting the average expression of a reference gene set. These per-cell pathway activity scores were then used as input features for a logistic regression classifier. This comparison setup parallels our evaluation of SDAN against other competing methods in Supplementary Section F. The results are summarized in Supplementary Table E.

Relative to this classical DE + enrichment baseline, SDAN achieved better performance in four of the five benchmark settings and remained competitive in the remaining transfer setting. Overall, these results suggest that SDAN captures phenotype-relevant structure more effectively than a conventional workflow that first identifies DE genes and then maps them to predefined pathway annotations. Although the classical baseline performed slightly better in the transfer setting, the overall pattern supports the advantage of SDAN in learning more predictive and task-specific gene programs across diverse cohorts.

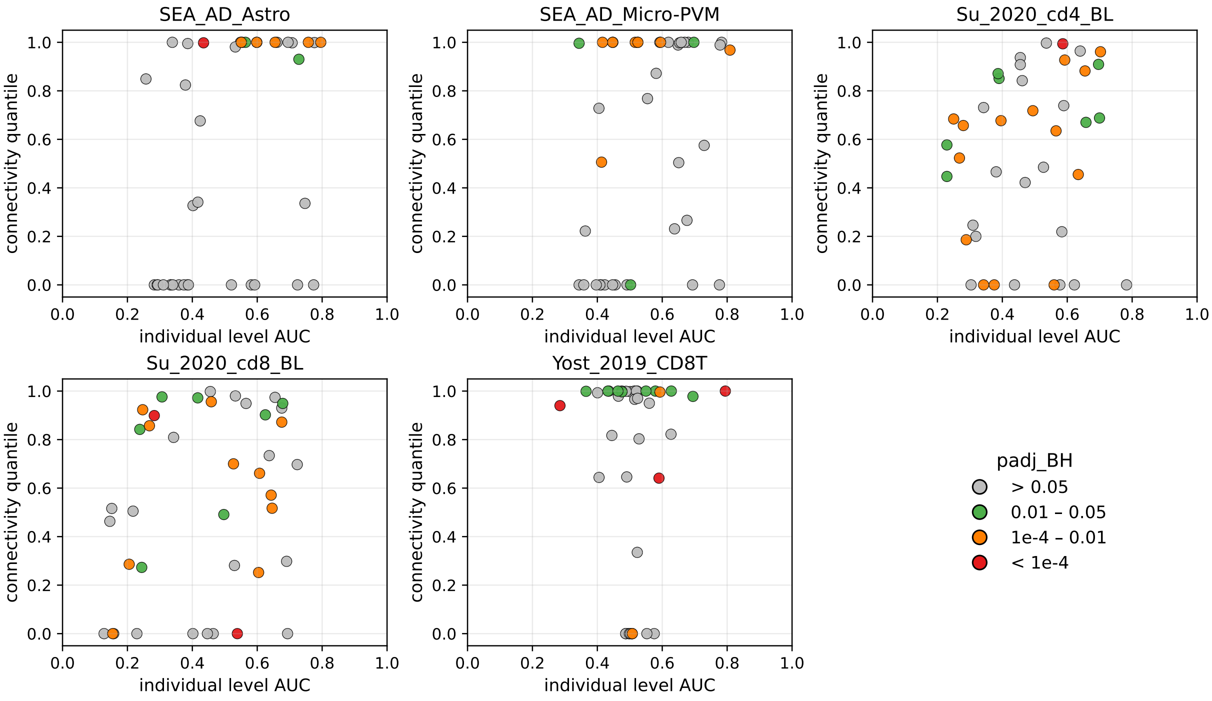

Figure J: Scatter plots of SDAN gene programs across five data sets. Each panel shows one data set: SEA-AD Astro, SEA-AD Micro-PVM, Su et al. (2020) $CD4^+$ T, Su et al. (2020) $CD8^+$ T, and Yost et al. (2019) $CD8^+$ T. Each dot represents one of the 40 SDAN programs; the x-axis shows the individual-level AUC and the y-axis shows the connectivity quantile. Dots are colored by the Benjamini–Hochberg-adjusted p-value (p_{adj}) of the hypergeometric overlap between the SDAN program's gene set and its best-matching curated baseline term (GO_BP, Reactome, or immune term). Gray: $p_{\mathrm{adj}} > 0.05$ (not significant); green: $0.01 < p_{\mathrm{adj}} \leq 0.05$; orange: $10^{-4} < p_{\mathrm{adj}} \leq 0.01$; red: $p_{\mathrm{adj}} \leq 10^{-4}$. The legend (shared across panels) is shown in the empty sixth grid cell.

Table E: Comparison of logistic regression AUC at the cell and individual levels (SDAN weight = 2.0). The best-performing result for each dataset and evaluation level is highlighted in red.

Method	Level	CD4+ T	CD8+ T	Astro	Micro-PVM	CD8+ T
SDAN	Cell	0.9015	0.8833	0.7193	0.6398	0.5380
	Individual	0.9524	0.9821	0.7482	0.7368	0.6786
Enrichment	Cell	0.7619	0.7685	0.6232	0.5186	0.5907
	Individual	0.8899	0.8988	0.7075	0.5760	0.6964

F Comparison of SDAN vs. Spectra, sciRED, and scNET

We first compared the gene programs identified by SDAN with those identified by sciRED (Supplementary Fig K) and Spectra (Supplementary Fig L). We did not include scNET in this comparison because scNET does not provide an explicit loading matrix. To quantify how well each SDAN gene program is recovered by sciRED or Spectra, we compared the corresponding loading vectors. For each SDAN gene program, the genes assigned to that program were treated as positives and all remaining genes as negatives. Using the loading vector for each gene program from sciRED or Spectra, we computed an AUC for every pair consisting of one SDAN gene program and one sciRED or Spectra gene program. For each SDAN gene program, we then retained the best-matching sciRED or Spectra gene program, defined as the one with the highest AUC.

Using the common downstream logistic regression framework described in the main paper, we compared the classification accuracy of SDAN with Spectra, sciRED, and scNET across the three datasets summarized in Supplementary Table F. Overall, SDAN achieved competitive or best-performing results in all datasets, particularly for COVID-19 severity prediction, where it gave the highest individual-level AUC for both CD4+ T cells (0.9524) and CD8+ T cells (0.9821), while remaining comparable to the alternative methods in the dementia and immunotherapy analyses. At the same time, the qualitative structure of the inferred gene programs differed substantially across methods. As shown in Supplementary Fig M, SDAN produced sparse and well-separated loading patterns for both astrocytes and microglia, with most genes loading strongly on only a small number of programs. In contrast, sciRED yielded substantially denser loading matrices with more diffuse signal across programs (Supplementary Fig N), making the resulting programs more difficult to interpret as discrete gene sets. Spectra showed an intermediate degree of sparsity, but its loading structure remained less sharply defined than that of SDAN (Supplementary Fig O). Taken together, these results support the main conclusion that SDAN retains predictive performance comparable to or better than competing methods while providing more interpretable and functionally coherent gene programs.

In Supplementary Figs K and L, each point represents one SDAN gene program. The x-axis shows the loading-vector AUC for the best-matching gene program from sciRED or Spectra, and the y-axis shows the graph-connectivity quantile, computed in the same way

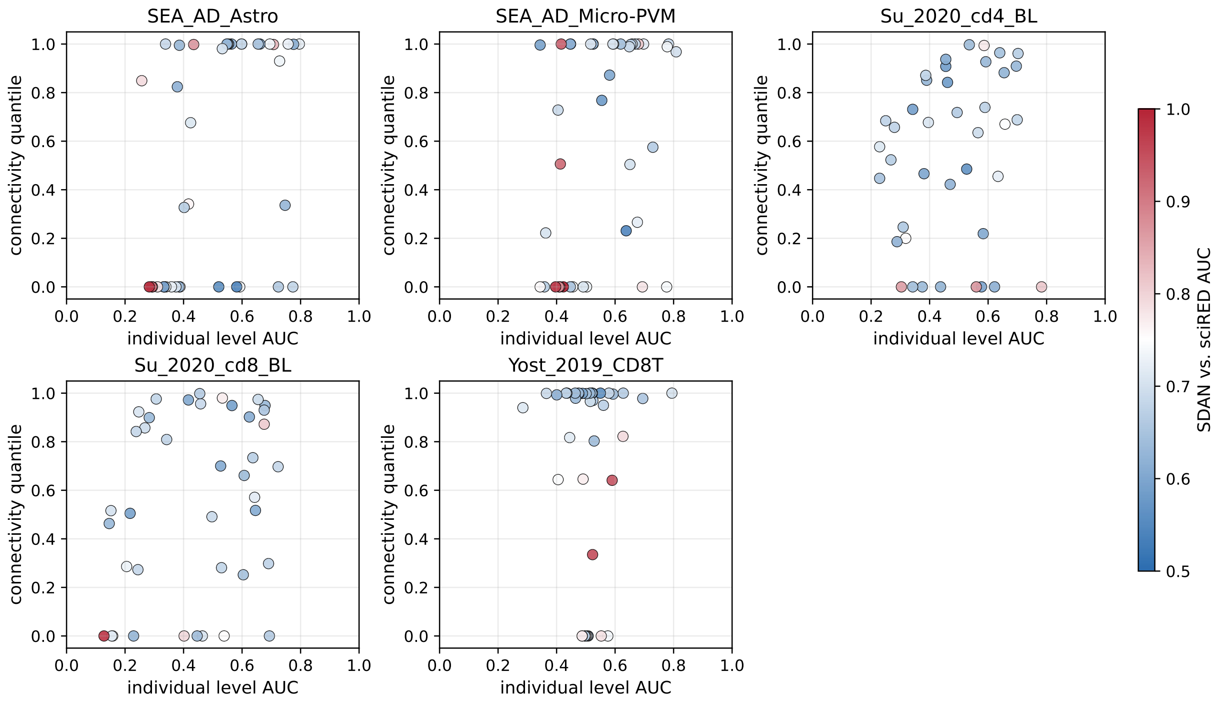

Figure K: Comparison between SDAN gene programs and their best-matching sciRED gene programs based on loading-vector AUC. Each point represents one SDAN gene program.

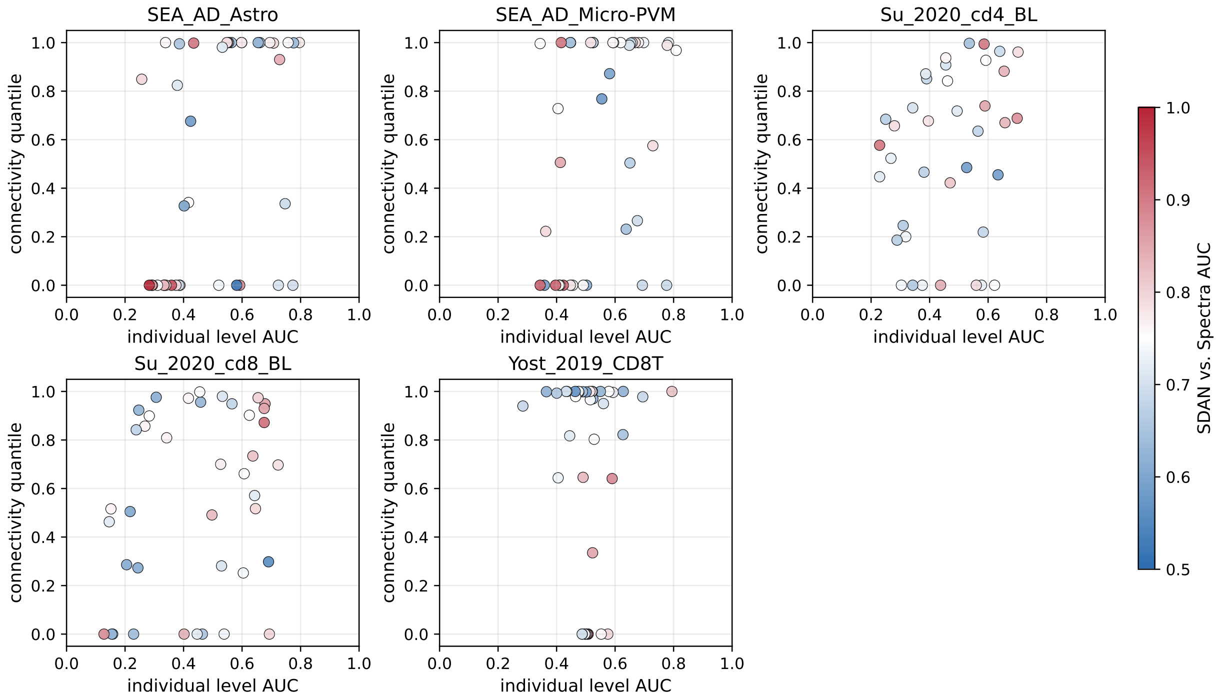

Figure L: Comparison between SDAN gene programs and their best-matching Spectra gene programs based on loading-vector AUC. Each point represents one SDAN gene program.

as in Fig 5. Points are colored according to the individual-level AUC obtained from the corresponding SDAN gene program on the test data. For both figures, the overall pattern is similar: SDAN identifies a subset of gene programs with very high graph-connectivity quantiles, but these programs are only imperfectly recovered by the alternative methods. Many points lie near the top of the plots, indicating that SDAN has learned gene programs that are strongly supported by the gene annotation graph, whereas the corresponding best-match AUC values are often only moderate rather than close to 1. This suggests that sciRED and Spectra capture some overlap with SDAN's gene programs, but neither method fully reproduces many of SDAN's most functionally coherent gene sets. Overall, these results indicate that SDAN learns gene programs that are more structurally coherent and more distinctly defined than those inferred by sciRED or Spectra, even when the alternative methods exhibit partial agreement.

Table F: Comparison of logistic regression AUC at the cell and individual levels (SDAN weight = 2.0). The best-performing setting for each dataset and evaluation level is highlighted in red.

Method	Level	CD4+ T	CD8+ T	Astro	Micro-PVM	CD8+ T
SDAN	Cell	0.9015	0.8833	0.7193	0.6398	0.5380
	Individual	0.9524	0.9821	0.7482	0.7368	0.6786
Spectra	Cell	0.8032	0.7696	0.7788	0.6832	0.5424
	Individual	0.9315	0.9256	0.7324	0.7664	0.5357
sciRED	Cell	0.8999	0.8838	0.7556	0.6620	0.5141
	Individual	0.9494	0.9583	0.7483	0.7460	0.6429
scNET	Cell	0.8514	0.8333	0.6983	0.6636	0.6080
	Individual	0.9464	0.9196	0.7551	0.7506	0.6786

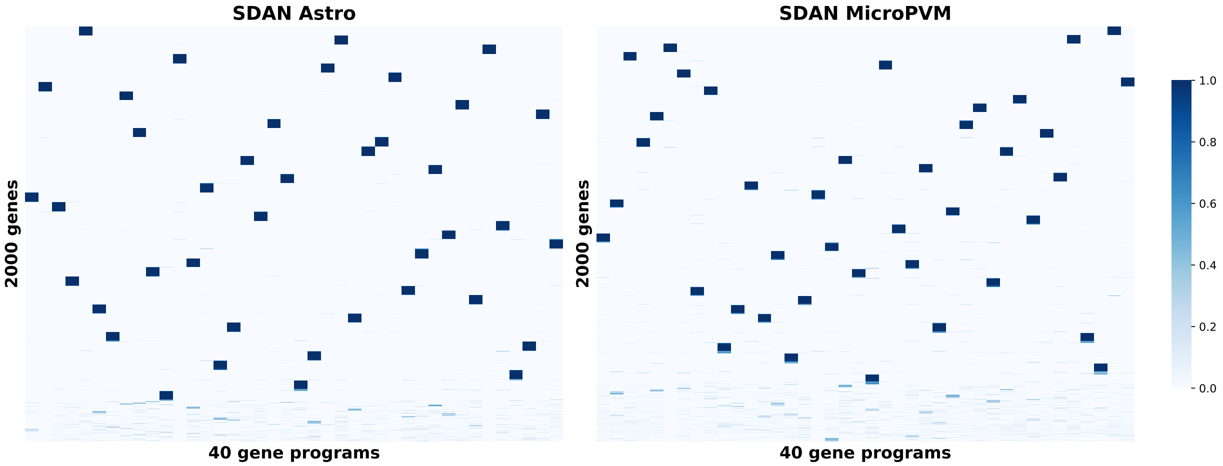

Figure M: Gene loading matrices inferred by SDAN from astrocyte and microglia gene expression data.

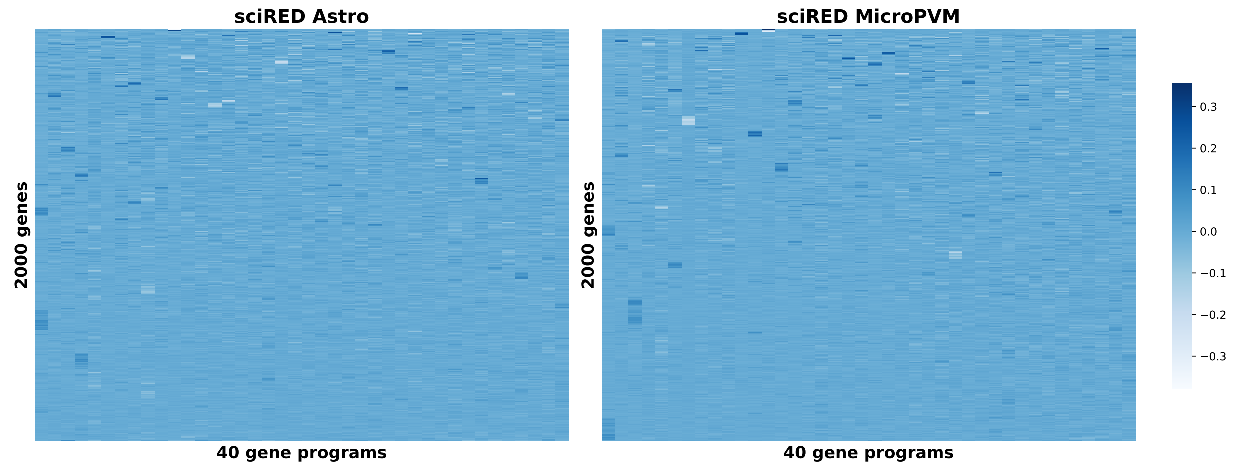

Figure N: Gene loading matrices inferred by sciRED from astrocyte and microglia gene expression data.

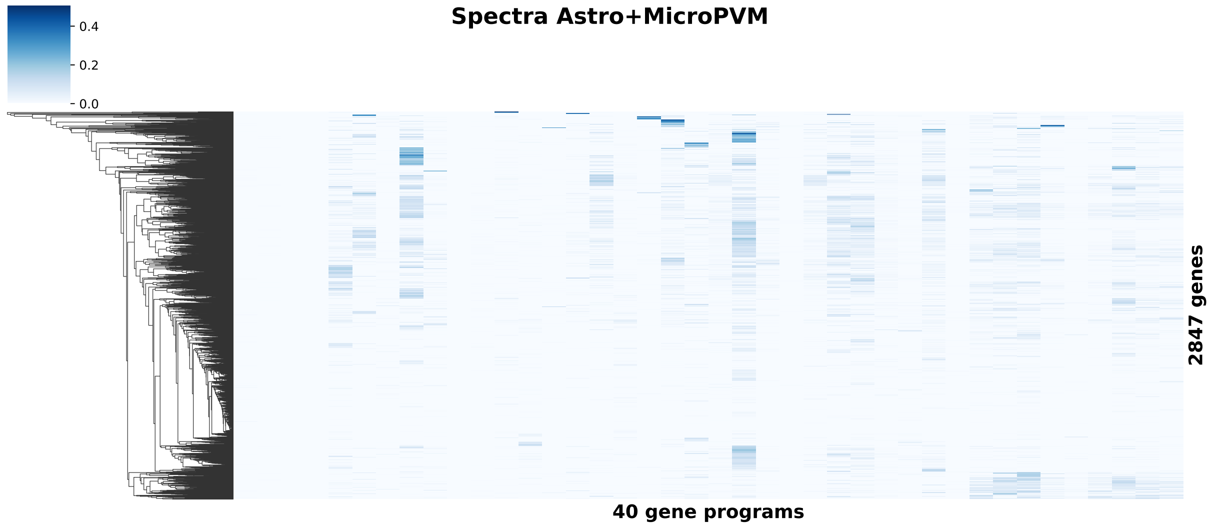

Figure O: Gene loading matrices inferred by Spectra from astrocyte and microglia gene expression data.

References

Gabitto, M. I., Travaglini, K. J., Rachleff, V. M., Kaplan, E. S., Long, B., Ariza, J., Ding, Y., Mahoney, J. T., Dee, N., Goldy, J., et al. (2024). Integrated multimodal cell atlas of alzheimer's disease. *Nature neuroscience*, 27(12):2366–2383.

Liberzon, A., Birger, C., Thorvaldsdóttir, H., Ghandi, M., Mesirov, J. P., and Tamayo, P. (2015). The molecular signatures database hallmark gene set collection. *Cell systems*, 1(6):417–425.

Sade-Feldman, M., Yizhak, K., Bjorgaard, S. L., Ray, J. P., de Boer, C. G., Jenkins, R. W., Lieb, D. J., Chen, J. H., Frederick, D. T., Barzily-Rokni, M., et al. (2018). Defining T cell states associated with response to checkpoint immunotherapy in melanoma. *Cell*, 175(4):998–1013.

Su, Y., Chen, D., Yuan, D., Lausted, C., Choi, J., Dai, C. L., Voillet, V., Duvvuri, V. R., Scherler, K., Troisch, P., et al. (2020). Multi-omics resolves a sharp disease-state shift between mild and moderate covid-19. *Cell*, 183(6):1479–1495.

Yost, K. E., Satpathy, A. T., Wells, D. K., Qi, Y., Wang, C., Kageyama, R., McNamara, K. L., Granja, J. M., Sarin, K. Y., Brown, R. A., et al. (2019). Clonal replacement of tumor-specific T cells following PD-1 blockade. *Nature medicine*, 25(8):1251–1259.

Young, M. D., Wakefield, M. J., Smyth, G. K., and Oshlack, A. (2010). Gene ontology analysis for RNA-seq: accounting for selection bias. *Genome biology*, 11:1–12.

Zheng, G. X., Terry, J. M., Belgrader, P., Ryvkin, P., Bent, Z. W., Wilson, R., Ziraldo, S. B., Wheeler, T. D., McDermott, G. P., Zhu, J., et al. (2017). Massively parallel digital transcriptional profiling of single cells. *Nature Communications*, 8(1):14049.
