# Supplementary material for: Supervised deep learning with gene functional annotation for cell classification
Source: PLoS Comput Biol. 2026 Jun 1;22(6):e1014327. doi: 10.1371/journal.pcbi.1014327 (PMC13235940; doi:10.1371/journal.pcbi.1014327)
Supplement: Attachment — Submitted filename: Review.pdf [file pcbi.1014327.s002.pdf]

This manuscript proposes SDAN, a supervised deep learning framework that integrates a gene-gene annotation graph with scRNA-seq expression (via a graph neural network) to learn interpretable gene sets that give cell-level scores, which are then aggregated to support individual-level prediction across three case studies (COVID-19 severity, dementia status, and immunotherapy response). My main concern is that the paper's positioning and practical value proposition are not yet fully clear: is SDAN primarily a predictive model, or a gene-set discovery/interpretation tool for understanding group differences? Several key baselines and robustness analyses are needed to support claims of interpretability, general utility, and biological insight.

Major issues

1. Clarify the primary objective and claims (prediction vs interpretation).
The manuscript alternates between emphasizing (i) improved classification/prediction and (ii) interpretable gene sets explaining group differences. What is the major output of SDAN?

2. Add standard, widely used baseline workflows for group comparison.
Since the motivating pain point is interpretability of gene-by-gene DE results, it is important to compare SDAN to a “classic” pipeline: DE (e.g., Wilcoxon/t-test) between groups (e.g., severe vs mild), followed by GSEA / GO / pathway enrichment for interpretation.
Without this, it is hard to justify why a user should adopt a fixed number of learned gene sets rather than letting the data define DE genes and enriched pathways.

3. Benchmark against common gene program / module discovery approaches (beyond sciRED/Spectra).
If “interpretable gene sets” are a key selling point, please compare (at least qualitatively, ideally quantitatively) to widely used gene-module approaches such as NMF-based gene programs (e.g., geneNMF/cNMF-like frameworks). The key question is: *what does SDAN add in interpretability or biological insight beyond established module discovery methods?*

4. Consistency and quality of selected genes/gene sets across methods.
When comparing methods, it is not clear whether the identified genes/gene sets overlap substantially across approaches, and whether SDAN preferentially selects more biologically meaningful or reproducible gene sets. Including quantitative overlap metrics and qualitative examples would strengthen the comparison

5. Guidance for weight/parameter tuning.
The manuscript should provide practical guidance on how users should tune the weighting scheme for new datasets (or always choose a weight of 2? Which seems impractical). In particular, how should users assess whether a chosen weight is appropriate when ground-truth phenotypes are unavailable or uncertain?

6. Dependence on the provided gene–gene network/annotation.
To what extent are SDAN’s prediction and interpretability results sensitive to the input gene–gene network (or annotation resource)? Please discuss potential biases introduced by the network prior, and whether important genes could be underweighted or missed if the predefined gene sets perform poorly for a given dataset.

7. Clarify phenotype/generalization scope (binary only?).
Please state clearly whether SDAN supports multi-class or continuous outcomes, and if so, provide either a demonstration or implementation details (loss functions, evaluation metrics, aggregation strategy).

8. Real-data case study design: cell-type inclusion and aggregation choices need stronger justification.

- COVID-19: Why restrict to CD4/CD8 T cells when other lineages (e.g., myeloid compartments) are often central to severity? If the goal is individual-level prediction, please justify the cell-type selection and consider an analysis using a broader set of immune cell types. Also clarify whether aggregation weights cell types equally or implicitly weights by cell counts.
- Alzheimer’s disease: Similarly, please justify focusing on only two cell types for a highly complex phenotype, and discuss whether incorporating more profiled cell types changes conclusions or improves performance.
- Immunotherapy response: The highlighted mitochondrial-associated gene set may be confounded by scRNA-seq QC decisions (e.g., % mitochondrial filtering) and may reflect stress/apoptosis rather than clinically actionable mechanisms. I strongly recommend adding confounder checks (QC covariates, stress signatures) and consulting domain clinicians to ensure the biological interpretation is appropriate and not a technical artifact.

Minor issues

1. Terminology: "Gene annotation" is used to mean a gene-gene interaction/knowledge graph, but "annotation" can mean many things in genomics. Please define it clearly at first use and specify what sources/edge types are included.
2. Figure organization: The ordering/appearance of Figure 2 panels is confusing (e.g., 2A/2B appearing after later panels). Please reorder or revise references to improve readability.
