# Supplementary material for: Supervised deep learning with gene functional annotation for cell classification
Source: PLoS Comput Biol. 2026 Jun 1;22(6):e1014327. doi: 10.1371/journal.pcbi.1014327 (PMC13235940; doi:10.1371/journal.pcbi.1014327)
Supplement: Attachment — Submitted filename: SDAN_responses.pdf [file pcbi.1014327.s004.pdf]

Response to the Review of PCOMPBIOL-D-26-00188

We thank the Additional Editor and the Reviewers for the careful reading of our manuscript and for the constructive suggestions. In response, we substantially revised both the manuscript and the supplementary material. The main changes are: (i) we clarified that SDAN is primarily a supervised gene-program discovery method rather than a purely predictive black-box classifier; (ii) we added additional benchmark analyses, including scNET and a conventional DE+Enrichment workflow; (iii) we added sensitivity analyses for gene preselection and dependence on the external gene annotation graph; (iv) we strengthened the discussions on some practical issues of SDAN; and (v) we improved terminology, figure clarity, metric definitions, and code reproducibility.

Additional Editor Comments:

Your manuscript has been assessed by our reviewers who are experts in the field. Based upon these reviews, we would be willing to consider a revision. You would need to compare and evaluate the proposed method with both standard benchmark methods and more recently proposed method such as scNET. In addition, please address reviewers' comments on clarification of the primary objective, filtering prior to GNN, and interpretation of real data analysis results.

We thank the Additional Editor for this clear guidance. In the revision, we addressed all four points directly. First, we added benchmark comparisons against scNET and against a conventional DE+Enrichment workflow. Second, we revised the Abstract, Introduction, and Discussion to clarify that SDAN is primarily a supervised gene-program discovery method whose main output is a set of phenotype-associated, interpretable gene programs (or gene sets); prediction accuracy serves as part of the loss function so that the learned gene programs are relevant to the phenotype. Third, we added a sensitivity analysis examining how the gene pre-selection step affects the learned gene programs and downstream performance. Fourth, we revised the real-data sections with clarifications and interpretations, including the justification on the selected cell types, and the explanation of that the mitochondrial gene program are not due to quality control issues because they are not genes from the mitochondrial chromosomes.

We have highlighted all the changes in red in the revised manuscript and provided point-by-point response below.

Reviewer 1

The manuscript introduces Supervised Deep learning with gene ANnotation (SDAN), a graph neural network (GNN)-based framework designed for the supervised classification of cells and individuals

using single-cell RNA-sequencing (scRNA-seq) data. The core motivation is to move beyond traditional gene-by-gene differential expression (DE) analysis—which often yields long, uninterpretable lists of genes with small effect sizes—toward identifying functionally coherent gene sets that directly optimize classification performance. SDAN integrates gene expression profiles with gene-gene interaction networks (e.g., BioGRID) and employs a graph pooling operation to cluster genes into latent components. These components serve as "gene sets" used to classify cell states (e.g., severe vs. mild COVID-19) and are subsequently aggregated to provide individual-level clinical predictions. My comments are listed below:

Thank you for reviewing our paper and provide insightful and constructive comments. We have highlighted all the changes in red in the revised manuscript and provided point-by-point response below.

Major:

1. SDAN has a similar design as scNET (Ron Sheinin et al. Nature Methods. 2025): both using scRNA-seq data and gene-gene interaction as input into a graph neural network for better functional annotation of scRNA-seq across cell types or biological conditions. The authors should compare scNET with SDAN, and discuss the strengths and weaknesses of each methods.

We appreciate the reviewer's suggestion to compare our approach with scNET. While both SDAN and scNET integrate scRNA-seq expression data with gene–gene interaction networks, their modeling goals and outputs are different. scNET is primarily a *representation learning* framework that learns network-aware embeddings for genes and cells by jointly modeling expression profiles and protein–protein interaction (PPI) networks. The resulting latent representations are useful for exploratory analyses such as cell clustering, pathway activity inference, or perturbation analysis. In contrast, SDAN is designed as a *supervised gene program learning* framework whose primary objective is to identify interpretable gene programs (or gene sets) that are optimized for functional annotation consistency and accuracy of phenotype prediction. Specifically, SDAN learns a sparse gene loading matrix that groups genes into biologically coherent gene programs using a graph neural network, and then uses these gene programs as features for downstream classification.

Consequently, the two methods serve complementary purposes. scNET excels at learning flexible, network-informed embeddings that capture complex relationships between genes and cells in an unsupervised setting. However, scNET itself does not generate gene programs or gene sets. In contrast, SDAN explicitly produces interpretable gene programs as part of the products of the training process, making it particularly suitable for applications where the goal is to identify biologically meaningful gene programs associated with a phenotype.

Since scNET does not produce gene programs, we cannot compare it with other methods in terms of gene programs. However, we can use the cell embedding produced by scNET for cell-level prediction. In the revised manuscript, we have now added such comparisons with scNET alongside SDAN, Spectra, and sciRED across all three real-data applications in Supplementary Table 6. We have also included this table below (Table 1). ScNET can achieve similar prediction performance as other methods.

In addition, we have added scNET to the Introduction as a relevant functional-annotation-based method, included and revised the comparison section to discuss both predictive performance and

Table 1: Comparison of logistic regression AUC at the cell and individual levels (SDAN weight = 2.0). The best-performing setting for each dataset and evaluation level is highlighted in red.

Method	Level	CD4+ T	CD8+ T	Astro	Micro-PVM	CD8+ T
SDAN	Cell	0.9015	0.8833	0.7193	0.6398	0.5380
	Individual	0.9524	0.9821	0.7482	0.7368	0.6786
Spectra	Cell	0.8032	0.7696	0.7788	0.6832	0.5424
	Individual	0.9315	0.9256	0.7324	0.7664	0.5357
sciRED	Cell	0.8999	0.8838	0.7556	0.6620	0.5141
	Individual	0.9494	0.9583	0.7483	0.7460	0.6429
scNET	Cell	0.8514	0.8333	0.6983	0.6636	0.6080
	Individual	0.9464	0.9196	0.7551	0.7506	0.6786

interpretability. We also added an implementation paragraph in the Methods section describing how scNET was trained in our experiments (on page 14 of the revised manuscript). In the Results and Discussion, we now clarify that although scNET and SDAN both integrate scRNA-seq data with gene–gene interaction networks, scNET is primarily an unsupervised representation-learning method that outputs cell embeddings, whereas SDAN is designed to learn sparse, phenotype-relevant, and directly interpretable gene programs through an explicit gene loading matrix. We further note that this difference makes scNET useful as a strong network-informed baseline, while SDAN is better suited for applications where the main goal is to identify biologically coherent and interpretable gene programs associated with the phenotype of interest.

2. The authors state that they pre-select marker genes using a one-sided Mann-Whitney U test to facilitate training. While this reduces computational overhead, it may introduce a "winner's curse" where the model only builds gene sets around genes that already show strong individual separation. A key promise of GNNs is the ability to pick up on subtle, coordinated signals from connected nodes. The authors should discuss whether SDAN can identify meaningful gene sets if pre-selection is relaxed, or provide a sensitivity analysis on how the pre-selection threshold affects the discovery of novel biology.

We thank the reviewer for raising this important point. The pre-selection step was introduced primarily to reduce computational cost and noise when training on very high-dimensional gene expression data, rather than to restrict the biological signals that SDAN can detect. In particular, a single-cell dataset often contains tens of thousands of genes, many of which are extremely sparse or uninformative, and training graph-based models on the full set of genes can substantially increase memory usage and training time.

Importantly, the SDAN framework is designed to capture coordinated signals across connected genes through the graph aggregation mechanism. As a result, the learned gene programs are not restricted to genes with strongest marginal effects. Even when pre-selection is used, SDAN can identify gene sets whose collective behavior across the graph provides predictive signal, including genes that may not exhibit very strong marginal differential expression signals.

To address the reviewer's concern more directly, we have now added a sensitivity analysis for DE-

based gene selection by varying the FDR threshold used in the pre-selection step (Supplementary Section 4.2). This analysis is intended to assess how strongly the candidate-gene screen affects downstream prediction and the biological interpretation of the learned gene sets. Across the range of FDR cutoffs we examined, prediction performance remained stable, and the learned gene sets were robust. These results indicate that while DE-based pre-selection defines the search space and therefore can influence which genes are ultimately available to SDAN, the main biological conclusions are robust to the choice of the threshold.

We have also revised the main text and added the following summary to the "Implementation of SDAN" subsection in Methods Section (on page 13 of the revised manuscript):

> We emphasize that this DE analysis is used only as a preselection step to reduce the original high-dimensional gene space to a manageable size for model training. The preselection is intentionally inclusive: after identifying DE genes for each class, we retain up to 1,000 highly variable marker genes per class and merge these sets across classes to construct a relatively large candidate gene pool. The final gene programs and gene sets are then learned by SDAN itself. Accordingly, genes that pass this initial filter may still receive negligible weights in the learned gene programs. To assess the sensitivity of SDAN to this preselection step, we varied the FDR threshold used for DE-based gene selection and reran SDAN under each setting. The results indicate that, even when more genes are selected using more liberal DE thresholds, a similar number of genes are ultimately included in the SDAN gene programs and the results remain robust to the DE threshold (Supplementary Section 4.2).

3. While the source code for SDAN is provided on GitHub, I encountered difficulties in creating a functional runtime environment due to unresolved dependency conflicts. To ensure the method is accessible to the broader research community, I strongly recommend that the authors distribute SDAN as a formal Python package (e.g., installable via pip or conda). At a minimum, the authors must provide a comprehensive requirements.txt or environment.yml file with strictly pinned versions for all dependencies to ensure long-term reproducibility.

We thank the reviewer for identifying this reproducibility issue. We have updated the repository to include a fully specified environment configuration with pinned dependency versions (*requirements.txt*) to ensure that the code can be reproduced reliably. We also provided detailed installation instructions and verified that the environment can be recreated on a clean system.

Minor:

1. The term "gene annotation" is inappropriately used. According to Ensembl, Gene annotation is the plotting of genes onto genome assemblies, and indexing their genomic coordinates (`http://useast.ensembl.org/info/genome/genebuild/index.html`). In the manuscript, it will be better described as "gene-gene interaction", "protein-protein interaction", or similar terms.

Thank you! We have changed the term to gene-gene interaction or "functional annoation" to avoid confusion.

2. For figure 5, "Hoyer sparsity" is described in the legend, but "hyper sparsity" is shown in the figure. Are they the same thing? In addition, the evaluation metrics, including Hoyer sparsity and quantiles of edges per component, should be clearly defined in the main Methods section, and

explained why these metrics indicate better performance.

We applologize for the typo. They should be Hoyer sparsity and we have fixed them. We have defined the evaluation metrics in the main Methods section (on page 11 of the revised manuscript).

3. The authors compared SDAN, Spectra, and sciRED across multiple datasets. It would be beneficial to include a direct comparison of the "functionally coherent" gene sets found by each method for the same biological process (e.g., show the gene weights of a biological pathway known to be involved in COVID-19, dementia or immunotherapy in all three methods) to demonstrate the "unambiguous assignment" claimed in the abstract.

Thank you for your suggestion. We have included this comparison in the revised manuscript, in Supplementary Materials Section 6, "Comparison of SDAN vs. Spectra, sciRED, and scNET". Because scNET does not provide gene programs, we compare SDAN with sciRED and Spectra. Since the gene programs inferred by sciRED and Spectra are not sparse, we evaluate whether SDAN gene sets tend to receive high loadings in the corresponding sciRED or Spectra gene programs using loading-vector AUC. As shown in Supplementary Figures 11 and 12. some SDAN gene programs have high loading-vector AUCs and thus have large overlap with sciRED or Spectra gene programs. There are also a number of SDAN gene programs with high graph connectivity (i.e., high consistency with functional annotation graph) and high individual-level AUC (indicated by the color scale), but moderate or low loading-vector AUCs. Therefore, sciRED and Spectra miss these important gene programs identified by SDAN.

Reviewer 2

This manuscript proposes SDAN, a supervised deep learning framework that integrates a gene–gene annotation graph with scRNA-seq expression (via a graph neural network) to learn interpretable gene sets that give cell-level scores, which are then aggregated to support individual-level prediction across three case studies (COVID-19 severity, dementia status, and immunotherapy response).

My main concern is that the paper's positioning and practical value proposition are not yet fully clear: is SDAN primarily a predictive model, or a gene-set discovery/interpretation tool for understanding group differences? Several key baselines and robustness analyses are needed to support claims of interpretability, general utility, and biological insight.

We appreciate your comments and agree that the original manuscript was not sufficiently clear on this critical point. We have revised the manuscript to clarify that SDAN is primarily a gene set (or gene program) discovery/interpretation tool for understanding group differences. Its main output is a collection of gene sets, and these gene sets can be used to predict the phenotype label of each cell. We have used a combination of a supervised prediction loss and a graph loss to discovery such gene sets. The prediction loss steers gene set discovery toward phenotype-relevant gene sets. The graph loss encourages genes that share similar annotations to be included in one gene set. We have revised the Abstract, Introduction, and Discussion accordingly. We have highlighted all the changes in red in the revised manuscript and provided point-by-point response below.

Major issues

1. Clarify the primary objective and claims (prediction vs interpretation). The manuscript alternates between emphasizing (i) improved classification/prediction and (ii) interpretable gene sets explaining group differences. What is the major output of SDAN?

Thank you for raising this important point that we need to clarify. The major output of SDAN is the "interpretable gene sets explaining phenotype-associated group differences". Although we evaluate the results of SDAN versus other methods, we do consider both prediction accuracy and interpretability, our primary objective is not to build a black-box classifier that maximizes prediction alone, but to identify functionally coherent and phenotype-relevant gene programs (or gene sets). In other words, prediction is the supervisory signal that guides gene program discovery, while the scientifically central output is the interpretable gene programs associated with the phenotype.

We have revised the manuscript to clarify the primary objective of SDAN. Specifically, we have added the following paragraph around the end of "Overview of SDAN" section (on page 3, in the second-to-last paragraph of the Overview section.):

> We emphasize that the primary goal of SDAN is to learn interpretable gene programs that are relevant to a phenotype of interest, rather than to optimize prediction accuracy alone. In SDAN, classification provides the supervisory signal that selects gene programs associated with the outcome, whereas gene annotation and sparsity constraints ensure that these gene programs remain biologically coherent and interpretable. Accordingly, the main output of SDAN is a set of phenotype-relevant gene programs, and the prediction scores should be viewed as a downstream summary derived from this gene program representation.

We have also added the following clarifications to the end of the "Discussion" section (on page 9 of the revised manuscript):

> Accordingly, SDAN should be viewed primarily as a method for discovering phenotype-relevant gene programs, with phenotype predictive accuracy serving as part of the loss function rather than the sole endpoint.

2. Add standard, widely used baseline workflows for group comparison. Since the motivating pain point is interpretability of gene-by-gene DE results, it is important to compare SDAN to a "classic" pipeline: differential expression (e.g., Wilcoxon/t-test) between groups (e.g., severe vs mild), followed by GSEA / GO / pathway enrichment for interpretation.

Without this, it is hard to justify why a user should adopt a fixed number of learned gene sets rather than letting the data define DE genes and enriched pathways.

We thank the reviewer for this suggestion. We totally agree that this DE + enrichment method is a baseline approach that we should compare. We have added comparison with this approach in the revised manuscript. Specifically, we first identified DE genes using the same DE procedure as in SDAN, then performed enrichment analysis using Gene Ontology Biological Process, Reactome, and immune-related gene set, with BH FDR control and Jaccard filtering to reduce redundancy. We retained the top 40 nonredundant enriched terms to match the default number of SDAN gene programs, converted them into per-cell pathway activity features, and used these features in a logistic regression classifier. We have compared the SDAN gene programs with those functional

categories identified by DE + enrichment methods. The details and complete results are presented in Supplementary Section 5 and we have summarized the results at the end of "Overview of SDAN" section (page 3 of revised manuscript):

> We have also compared SDAN with a baseline method: differential-expression followed by functional category enrichment analysis. To implement this baseline, we first identified differentially expressed genes between groups, then performed functional category enrichment analysis to identify top 40 "baseline terms". See Supplementary Materials Section 5 for details. The majority of the SDAN gene programs (around 75%) do not have significant overlap with those baseline terms. The results remain similar when we focus on those more informative SDAN gene programs that classify phenotype well and have high connectivity in the gene-gene interaction graph (Supplementary Table 4). This suggests that the baseline terms miss many informative SDAN gene programs. In addition, we also compared classification accuracy. Across five benchmark settings, SDAN outperformed the classical DE + enrichment workflow in four settings and remained competitive in the fifth setting. Details and results are provided in Supplementary Section 5.

3. Benchmark against common gene program / module discovery approaches (beyond sciRED/Spectra). If "interpretable gene sets" are a key selling point, please compare (at least qualitatively, ideally quantitatively) to widely used gene-module approaches such as NMF-based gene programs (e.g., geneNMF/cNMF-like frameworks).

The key question is: what does SDAN add in interpretability or biological insight beyond established module discovery methods?

We thank the reviewer for this helpful suggestion. We agree that factor-based methods such as cNMF are widely used and should be considered in the comparison. Among those factor-based methods, we chose to compare with the more recent method sciRED, which is a computationally efficient factor-analysis framework for single-cell data. As described in the sciRED paper, sciRED outperforms alternative factorization methods (including NMF-based approaches) in combined interpretability and runtime efficiency. In this sense, sciRED is positioned as an advance over earlier generic factorization workflows for large-scale single-cell data.

Our manuscript shows that SDAN yields substantially more interpretable gene programs than sciRED while maintaining comparable predictive performance in downstream classification. More specifically, SDAN gene programs are more interpretable for two reasons. First, SDAN's objective function imposes sparsity on gene programs so that each gene program typically contains tens of genes. In contrast, sciRED or any NMF-based methods do not enforce sparsity on gene weights, and thus each gene program may involve thousands of genes, which makes the resulting gene programs difficult to interpret. Second, because SDAN incorporates functional annotations of genes in its objective function, the resulting genes in each gene programs tend to share functional similarities. In contrast, sciRED or NMF-based methods do not have such enrichment of functionally related genes.

In the revised manuscript, we have added clarification that sciRED is a representative method for factor-based methods, including NMF-based methods, and the limitations of sciRED shown in our comparison are also the limitations shared by NMF-based methods. Specifically, we replaced the

last paragraph of "Introduction" section (on page 2):

> More recently, sciRED was developed as a computationally efficient factorization-based framework for large-scale scRNA-seq data, and it outperformed alternative factorization methods (including NMF-based approaches) in combined interpretability and runtime efficiency [1].

We have also added to the first paragraph of "Comparison of SDAN vs. Spectra, sciRED, and scNET" section (on page 7 of the revised manuscript):

> SciRED is a representative example of factorization-based methods such as cNMF. The limitations of sciRED shown in the comparison are common features shared by all factorization-based methods: lack of sparsity in gene programs and lack of guidance from functional annotation of genes.

4. Consistency and quality of selected genes/gene sets across methods. When comparing methods, it is not clear whether the identified genes/gene sets overlap substantially across approaches, and whether SDAN preferentially selects more biologically meaningful or reproducible gene sets. Including quantitative overlap metrics and qualitative examples would strengthen the comparison.

Yes, we totally agree that this is an important and necessary comparison. In the revised manuscript, we added a new section (Supplementary Section 6) for both quantitative and qualitative comparisons of the gene programs identified by SDAN versus alternative methods.

First, we now explicitly compare SDAN with sciRED and Spectra at the level of inferred gene programs. Because scNET does not provide an explicit loading matrix, it does not permit a directly comparable gene-program-level overlap analysis. For sciRED and Spectra, we quantify overlap by treating the genes assigned to each SDAN gene program as positives and all remaining genes as negatives, and then using the loading vector of each sciRED/Spectra program to compute an AUC for every cross-method program pair. For each SDAN gene program, we retain the best-matching sciRED or Spectra program. These results are now shown in Supplementary Figs. 11 and 12. These new analyses show that although sciRED and Spectra sometimes achieve partial overlap with SDAN programs, many SDAN gene programs with high graph-connectivity quantiles (hence good consistency with known gene–gene interaction annotation) are only moderately recovered by the alternative methods. In other words, the gene programs learned by SDAN are not simply redundant with those identified by sciRED or Spectra. Rather, SDAN preferentially identifies gene programs that are more strongly supported by the annotation graph and are more sharply defined as coherent gene sets.

We have added the following text in the revised manuscript, page 8

> We have compared the gene gene programs reported by SDAN, sciRED, and Spectra. Since scNET did not produce an explicit loading matrix for gene programs, we did not include it in this comparison. Because the gene programs by sciRED and Spectra were weights across many genes, we compared them with SDAN gene sets using AUC. A higher AUC indicates those genes with higher weights are more likely to belong to a SDAN gene set. For each SDAN gene set, we found its best match with the highest AUC. Many SDAN gene sets with high classification accuracies and high graph connectivities

(hence better alignment of gene-gene interaction graph) did not have large overlap with any sciRED or Spectra gene programs (Supplementary Figures 11- 12), suggesting that SDAN identify additional and informative gene sets than sciRED or Spectra.

Second, we expanded the comparison of interpretability and structural quality across methods. In the revised manuscript, we show that sciRED produces substantially denser loading matrices, with many programs involving thousands of genes, which makes them difficult to interpret as discrete gene sets. Spectra yields sparser programs than sciRED, but its loading structure remains less well defined than that of SDAN. By contrast, SDAN produces sparse and well-separated loading patterns, and its inferred gene sets exhibit stronger within-set connectivity in the underlying gene–gene interaction annotation graph. These results are summarized in the revised manuscript and in Fig. 5, Supplementary Table 6, and Supplementary Figs. 13–15.

5. Guidance for weight/parameter tuning. The manuscript should provide practical guidance on how users should tune the weighting scheme for new datasets (or always choose a weight of 2? Which seems impractical).

In particular, how should users assess whether a chosen weight is appropriate when ground-truth phenotypes are unavailable or uncertain?

We thank the reviewer for raising this important point. The weighting parameter w controls the trade-off between the supervised classification loss and the unsupervised graph-structure regularization loss. In our experiments, we used $w = 2$ as a default because it provided a good balance across the datasets studied in this paper; however, we do not intend to suggest that this value is universally optimal.

For a new dataset, we recommend tuning w over a small grid, for example $w \in \{0, 0.25, 0.5, 1, 2, 5, 10\}$. For each value of w, users should evaluate both predictive performance on a validation set and the consistency of the inferred gene sets with the functional annotation given by a gene-gene network. For the latter, we have developed a diagnostic quantity in the manuscript: the connectivity quantile of the genes within a gene set, relative to randomly selected gene sets. If this quantile is high, it means the genes in the inferred gene set are more likely to connected in the gene-gene network than randomly selected genes. Since w is the weight for graph loss, a larger w leads to better coherence with the gene-gene network, and a smaller w leads to higher prediction accuracy. We recommend choosing the smallest w that achieves stable and high biological coherence, as well as relatively high prediction accuracy.

If phenotypes are uncertain but available, users can still tune w by combining noisy-label validation performance with label-free structure diagnostics. In this case, the highest prediction accuracy is limited due to noise in the labels. However, the relative scale of the loss from smaller w's to larger w's are still informative. If phenotypes are completely unavailable, SDAN is not applicable as a supervised learning method. It is possible to develop an unsupervised framework similar to SDAN while using gene expression similarity within gene sets as part of the loss function, though it is beyond the scope of SDAN.

We have added these clarifications in the "Neural network architecture and loss functions" subsection of Methods Section (page 11 of the revised manuscript):

In practice, w should be selected by balancing predictive performance and interpretabil-

ity. We recommend fitting SDAN over a grid of values, e.g., $w \in \{0, 0.25, 0.5, 1, 2, 5, 10\}$, and evaluating each fit using both validation performance and structural diagnostics of the inferred gene programs. Useful diagnostics include the within-program connectivity quantile relative to randomly selected gene sets of the same size, the number of non-empty gene programs, and the functional enrichment of the inferred gene sets. We recommend selecting the smallest w for which validation performance remains close to the best observed value and these graph-based diagnostics have stabilized. When phenotype labels are available but potentially noisy, the absolute level of prediction accuracy may be limited by label uncertainty. In this setting, however, validation performance still provides useful relative information across values of w, especially when interpreted together with the graph-based diagnostics described above.

6. Dependence on the provided gene–gene network/annotation. To what extent are SDAN's prediction and interpretability results sensitive to the input gene–gene network (or annotation resource)? Please discuss potential biases introduced by the network prior, and whether important genes could be underweighted or missed if the predefined gene sets perform poorly for a given dataset.

Thank you for raising this important point. The gene–gene interaction network used by SDAN is a prior that encourages biologically coherent gene programs, but it is not imposed as a hard constraint. Instead, SDAN optimizes a supervised classification objective together with a graph-based regularization term, $L_{\text{classification}} + wL_{\text{graph}}$. The graph term encourages genes connected in the annotation network to be grouped into coherent programs, whereas the supervised term favors gene programs that improve prediction accuracy.

Because the graph enters only through a weighted regularization term, SDAN is not forced to follow an incorrect or incomplete annotation resource. In particular, the tuning parameter w controls the extent to which the model relies on the prior. If the annotation graph is informative, a moderate value of w can improve the biological coherence of the learned programs. If the graph is less informative for a given dataset, genes that are strongly predictive but weakly connected in the prior network can still receive substantial loadings through the supervised component of the model, rather than being excluded simply because they are poorly annotated.

We agree, however, that the network prior can introduce bias in the interpretability layer of the model. Well-annotated pathways or densely connected regions of the graph may be preferentially favored, whereas biologically important but weakly annotated genes may be underweighted in the learned gene programs. To assess this directly, we added a controlled graph-perturbation analysis in Supplementary Section 4.1, where we fixed the data, DE gene set, and model hyperparameters, and progressively rewired a fraction of BioGRID edges while preserving the total number of edges. This design isolates the effect of the annotation graph itself from changes in the input gene set.

The new results support two conclusions. First, SDAN's predictive performance is relatively robust to substantial graph perturbation: the test AUC remains fairly stable across perturbation levels, indicating that classification does not rely rigidly on the annotation prior. Second, the learned loading matrix becomes less interpretable when the graph is degraded. More specifically, under graph perturbation, the loading matrix becomes less sparse and less well separated, with more diffuse and overlapping gene programs rather than the cleaner, more distinct programs obtained from the original graph. This is consistent with the increase in L_o and the sharp drop in Hoyer

sparsity reported in Supplementary Table 2 and Supplementary Fig. 8. In other words, when the prior graph is biologically meaningful, it helps SDAN recover sparse, coherent, and easier-to-interpret gene programs; when the graph is corrupted, SDAN can still preserve prediction accuracy, but it does so with a less structured and less interpretable representation.

In summary, the annotation resource mainly affects the biological coherence and interpretability of the learned gene programs, whereas prediction is more robust to such graph perturbation. In future work, it would be valuable to evaluate robustness across multiple annotation resources and to combine complementary network databases to reduce such bias.

We have added to the end of "Neural network architecture and loss functions" sub-section of Methods Section (page 11):

> A key advantage of SDAN is that the annotation graph is incorporated through a weighted graph-regularization term rather than being hard-coded into the model structure. As a result, SDAN can adaptively balance biological interpretability and predictive accuracy: when the prior is informative, it encourages sparse and functionally coherent gene programs, whereas when the prior is less informative, the supervised objective can still preserve classification performance. This flexibility distinguishes SDAN from methods that ignore prior knowledge and from approaches that rely more rigidly on predefined biological networks.
>
> To assess robustness to the annotation graph, we performed a graph-sensitivity analysis in which the annotation graph was progressively perturbed while keeping the data, selected genes, and model hyperparameters fixed. These results are reported in Supplementary Section 4.1 and show that test AUC remains relatively stable even under substantial graph perturbation, indicating that SDAN's predictive performance is not overly dependent on the exact annotation graph. In contrast, the structure of the learned loading matrix is more sensitive to graph quality: as the graph is increasingly perturbed, the learned gene programs become less sparse, less well separated, and more overlapping, indicating reduced interpretability. These findings suggest that a biologically meaningful annotation graph mainly improves the coherence and interpretability of the learned gene programs, whereas prediction remains comparatively robust even when the prior is degraded.

7. Clarify phenotype/generalization scope (binary only?). Please state clearly whether SDAN supports multi-class or continuous outcomes, and if so, provide either a demonstration or implementation details (loss functions, evaluation metrics, aggregation strategy).

Yes, we totally agree that it is important to clarify the phenotype/generalization scope. The current implementation of SDAN is limited to binary phenotypes. However, the SDAN framework is general and can accommodate multi-class or continuous outcomes by replacing the supervised loss function while keeping the graph-based regularization unchanged.

For multi-class phenotypes, the binary cross-entropy loss can be replaced by the standard softmax cross-entropy loss, where the final classifier outputs a probability vector over classes. Evaluation can then be performed using standard metrics such as AUC. The aggregation from cell-level predictions to the individual level remains unchanged: cell-level class probabilities can be averaged across cells belonging to the same individual to produce subject-level predictions.

For continuous phenotypes, the supervised objective can be replaced by a regression loss, such as mean squared error. The model then predicts a continuous score for each cell, which can again be aggregated at the individual level by averaging across cells. Performance can be evaluated using standard regression metrics such as mean squared error.

Importantly, the graph-based minCUT regularization and sparsity structure remain unchanged, meaning the SDAN architecture and interpretability properties are preserved under these alternative outcome types.

To clarify the generality of the framework, we revised the manuscript to explicitly describe how the supervised loss can be adapted for multi-class and continuous outcomes and include implementation details in the Methods section.

We have added a new "Extensions" sub-section in the Methods Section (page 12):

> Although the current implementation and applications considered in this paper focus on binary phenotypes, the SDAN framework is not restricted to binary outcomes. For multi-class phenotypes, the binary cross-entropy loss can be replaced with the standard softmax cross-entropy loss, with the classifier outputting a probability vector over multiple classes. For continuous outcomes, the supervised objective can be replaced with a regression loss such as mean squared error. In both cases, the graph loss used to learn gene programs remains unchanged. Aggregation from cell-level predictions to the individual level can be performed using the same strategy as in the binary setting, for example by averaging predicted scores or probabilities across cells from the same individual. This flexibility allows SDAN to be applied to a broad range of phenotype types while preserving the interpretability of the learned gene programs.

8. Real-data case study design: cell-type inclusion and aggregation choices need stronger justification.

COVID-19: Why restrict to CD4/CD8 T cells when other lineages (e.g., myeloid compartments) are often central to severity? If the goal is individual-level prediction, please justify the cell-type selection and consider an analysis using a broader set of immune cell types. Also clarify whether aggregation weights cell types equally or implicitly weights by cell counts.

Thank you for raising this important question. We have added the following text in the revised manuscript to justify why we focus on T cells (the beginning of page 4).

> It has been well established that COVID-19 vaccines can reduce the risk of severe COVID-19 disease. Vaccines induce antibodies and memory T cells to fight SARS-CoV-2 virus. While antibodies protect us against infection [2–4], it fails to Omicron variants of SARS-CoV-2 virus [5, 6]. Several recent studies show that prompt T cell responses protect us from severe COVID-19 [7–13]. These results, combined with the finding that vaccine-induced memory T cells cross-recognize SARS-CoV-2 variants from Alpha to Omicron [12] suggest that T cells are the key factors in preventing severe COVID-19. Therefore, we study the relationship between gene expression in T cells and the severity of COVID-19 disease.

We have also clarified that cell type proportions themselves can be informative predictors. However, because our study focuses on cell type-specific differential expression analysis, systematic modeling

of cell type proportions is beyond the scope of the present work. We have added the following text in the revised manuscript (on page 4).

> It is worth noting that, although our focus is on cell type–specific gene expression, cell type proportions themselves are also informative for predicting severe COVID-19. For example, severe disease is associated with myeloid expansion (e.g., neutrophils) and lymphocyte depletion, which are likely the consequences rather than causes of the severe COVID-19 [14]. This is because an ineffective or delayed T cell response may lead to over-activation of the innate immune system, causing tissue damage and severe COVID-19. In contrast, a timely and effective T cell response can clear the virus and thus prevent severe COVID-19.

Alzheimer's disease: Similarly, please justify focusing on only two cell types for a highly complex phenotype, and discuss whether incorporating more profiled cell types changes conclusions or improves performance.

Thank you for your suggestion. Very briefly, many research works in the past 10 to 20 years have shown that immune processes play a pivotal role in the pathogenesis of Alzheimer's disease. Microglia and astrocytes are the two types of residence immune cells in brain and they are both causally linked to the pathogenesis of Alzheimer's disease. Therefore we focus on these two cell types to identify potentially causal factors for Alzheimer's disease. We have added the following text in the revised manuscript to justify our choice of cell types (on page 5). Please note that the citation numbers are different between the main text and this response letter because not all citations used in the paper are used here.

> Alzheimer's disease, which is associated with the accumulation of amyloid beta plaques and hyperphosphorylated tau, is the most common cause of dementia in older individuals [15]. Immune processes play a pivotal role in the pathogenesis of Alzheimer's disease [16]. Microglia and astrocytes are the two types of residence immune cells in brain, and both have been causally linked to Alzheimer's disease pathogenesis [16–19]. Activated microglia have long been reported to cluster near plaques [20], and later studies established a causal mechanism linking microglia to synaptic degeneration [17]. Since then, many other studies have demonstrated the functional roles of microglia (or in general, neuroinflammation) in Alzheimer's disease [16], and the importance of microglia is further supported by findings from Genome-wide association studies [21]. Astrocytes have also long been established as an important neuroinflammation factor in Alzheimer's disease [18] and that neurotoxic astrocytes are induced by microglia [19]. Given the important and established causal roles of microglia and astrocytes in Alzheimer's study, we focus on these two cell types to study their cell type-specific expression.

Immunotherapy response: The highlighted mitochondrial-associated gene set may be confounded by scRNA-seq QC decisions (e.g., % mitochondrial filtering) and may reflect stress/apoptosis rather than clinically actionable mechanisms. I strongly recommend adding confounder checks (QC covariates, stress signatures) and consulting domain clinicians to ensure the biological interpretation is appropriate and not a technical artifact.

Thank you for your suggestion. We agree this is important to clarify. The gene expression abundance for genes that are located on mitochondrial chromosome is routinely used for scRNA-seq

QC. However, none of the genes in the gene set that we identified is located in the mitochondrial chromosome. The locations of these genes have been listed in Supplementary Table 1. This gene set captures mitochondrial translation and respiratory capacity, reflecting the metabolic fitness of T cells, which is a key determinant of their functional persistence and response to immune checkpoint blockade. We have added the following text in the revised manuscript (at the end of page 7).

> It is important to note that expression of genes located in the mitochondrial chromosome is often used as a quality-control criterion in scRNA-seq analysis, because in stressed and dying cells, the cytoplasmic mRNAs degrade faster than the mRNAs encoded by the mitochondria chromosomes. However, none of the genes in this SDAN-derived gene set is located in the mitochondrial chromosomes (Supplementary Table 1). Therefore, this mitochondrial signal is unlikely to reflect an underlying quality-control artifact.

Minor issues

1. Terminology.

"Gene annotation" is used to mean a gene–gene interaction/knowledge graph, but "annotation" can mean many things in genomics. Please define it clearly at first use and specify what sources/edge types are included.

Thank you. We have clarified the gene annotation as functional annotation of genes (e.g., protein-protein interaction) in both abstract and introuduction of the revised manuscript.

2. Figure organization.

The ordering/appearance of Figure 2 panels is confusing (e.g., 2A/2B appearing after later panels). Please reorder or revise references to improve readability.

Thank you. We have double checked that 2A/2B do appear at the top-left corner of Figure 2.

Reviewer 3

This study presents SDAN, a computational framework which incorporates graph neural network to learn gene set assignments for the classification of cell-level and patient-level metadata. While the manuscript evaluates SDAN on three datasets and benchmarks it against two existing methods, the presentation of the results could be strengthened to more clearly highlight the model's novelty and its performance advantages. Below are my detailed comments.

Thank you for your suggestions and constructive comments. We have revised our paper to better highlight the model's novelty and performance advantages. We have highlighted all the changes in red in the revised manuscript and provided point-by-point response below.

Major Comments:

1. The main motivation of this work is to solve the effect size issue of differential expression (DE) analysis arising from the large sample size typical of scRNA-seq data, where even genes with minimal biological effect can achieve extremely small p-values. To mitigate this, the study proposes learning gene set assignments based on the top DE genes identified. While this is an interesting

direction, it would be helpful to more clearly explain how this strategy substantively mitigates the stated issue. Specifically, because genes are initially selected based on statistical significance, genes with negligible biological relevance may still be maintained. Conversely, genes that do not meet the initial DE threshold are excluded entirely and cannot be reconsidered later, potentially introducing an irreversible selection bias. It would strengthen the manuscript to provide additional justification or empirical evidence demonstrating that the proposed framework meaningfully alleviates the effect size concern.

We thank the reviewer for this insightful comment. We agree that the initial differential-expression analysis should not be viewed as the final inferential step of SDAN and it is important to provide additional justifications and sensitivity studies when using different differential expression (DE) thresholds. We have revised our paper to add such justifications and empirical evidence.

The role of the initial screening step is very much limited: it is used only as an initial prescreening step to reduce the original gene space to a computationally manageable candidate set for model training. In our implementation, this screening is intentionally inclusive. After identifying DE genes for each class, we retain up to 1,000 highly variable marker genes per class and combine them across classes, so the candidate pool remains fairly large rather than being restricted to only a few top-ranked genes. For example, in the COVID-19 analyses, this yields 1,649 genes for CD8+ T cells and 2,000 genes for CD4+ T cells; in the dementia analyses, we retain 2,000 genes for each cell type.

More importantly, genes passing this prescreening step are not automatically retained in the final result. SDAN further selects and organizes genes through joint optimization of the supervised classification loss and the graph-based unsupervised loss. The learned loading matrix is encouraged to be sparse and close to binary, so genes that are not useful for prediction or not supported by the gene-gene interaction graph receive negligible contribution in the final gene sets. Therefore, the final reported gene sets are determined by this second-stage learning procedure, not by DE significance alone.

We also agree that any prescreening step may exclude genes with weak marginal signals but useful joint effects. However, because the prescreening is deliberately broad and followed by substantial downstream refinement by SDAN, we believe the resulting selection bias is limited in practice. In addition, our empirical results show that SDAN does more than preserve the initial DE list. Compared with competing methods, SDAN yields substantially sparser gene sets that align with gene-gene interaction graph. These results support that SDAN meaningfully mitigates the practical interpretability problem caused by large DE gene lists with weak individual effects.

In this revision, we conducted an additional sensitivity analysis to evaluate the genes selected by SDAN when using different DE thresholds for pre-screening. We found the total number of genes selected were similar across DE thresholds and the selected genes had significant overlap with each other. The complete results are presented in Supplementary Materials Section 4.2.

In the revised manuscript, we have added the following text in the “Implementation of SDAN” sub-section of Methods Section (on page 13) to justify the DE-based pre-screening and summarize the results of the sensitivity analysis.

> We emphasize that this DE analysis is used only as a preselection step to reduce the original high-dimensional gene space to a manageable size for model training. The

preselection is intentionally inclusive: after identifying DE genes for each class, we retain up to 1,000 highly variable marker genes per class and merge these sets across classes to construct a relatively large candidate gene pool. The final gene programs and gene sets are then learned by SDAN itself. Accordingly, genes that pass this initial filter may still receive negligible weights in the learned gene programs. To assess the sensitivity of SDAN to this preselection step, we varied the FDR threshold used for DE-based gene selection and reran SDAN under each setting. The results indicate when more genes are selected using more liberal DE thresholds, a similar number of genes are finally included in the SDAN gene programs and the results remain robust to the DE threshold (Supplementary Section 4.2).

2. Figure 2C&F, 3A-B & C-D show the cell-level and individual-level precision score respectively. However, Figure 2F and 3A&B's x-axis and y-axis is not labeled, which makes interpretation difficult. In addition, only the distribution of prediction scores is shown, while the classification performance is not presented. It would be more informative to report the classification metrics, such as AUC score, of SDAN and compare it to the benchmarking methods.

We appologize for the omission of axis labels. In the revised manuscript, we have added explicit x- and y-axis labels to Fig. 2(F) and Fig. 3(A-D). Specifically, for the cell-level histograms, the x-axis is now labeled as "Cell-level prediction score" and the y-axis as "Density". For the individual-level boxplots, the x-axis is now labeled as "Donor class" and the y-axis as "Ind. prediction score", indicating individual-level prediction score.

We also appreciate the reviewer's point regarding classification performance. AUC-based classification results were already reported in the main text for all three applications, including the COVID-19, dementia, and immunotherapy analyses, and the comparison with Spectra and sciRED was summarized in the benchmarking subsection, with full quantitative results provided in Supplementary Table 6.

3. The study reports gene set enrichment analysis results to demonstrate the biological relevance of the identified gene sets, it remains unclear how these gene sets are intended to replace or improve upon traditional DE analysis. The manuscript would benefit from a clearer explanation of how gene set–level outputs provide comparable or superior interpretability relative to standard DE results. Additionally, some gene sets may contribute to both classes. For example, mild and severe condition of COVID-19 may share similar pathway, but the degree of contribution can be different. Clarifying how SDAN captures and quantifies such differential contributions would enhance the interpretability claims of the framework.

We thank the reviewer for this suggestion. We added a standard DE + enrichment baseline in Supplementary Section 5. Specifically, we first identified DE genes using the same DE procedure as in SDAN, then performed enrichment analysis using Gene Ontology Biological Process, Reactome, and immune-related gene sets, with BH FDR control and Jaccard filtering to reduce redundancy. We retained the top 40 nonredundant enriched terms to match the default number of SDAN gene programs, converted them into per-cell pathway activity features, and used these features in a logistic regression classifier. This comparison directly addresses the concern that one could simply let DE genes and enriched pathways define the interpretation. We have compared the functional categories identified by SDAN with those identified by DE + enrichment methods. In addition, we also compared classification accuracy. Across five benchmark settings, SDAN outperformed the

classical DE + enrichment workflow in four settings and remained competitive in the fifth setting. The complete results are reported in the newly added Supplementary Section 5.

In the revised manuscript, we have added the following text at the end of "Overview of SDAN" section (on page 3 of revised manuscript):

> We have also compared SDAN with a baseline method: differential-expression followed by functional enrichment analysis. To implement this baseline, we first identified differentially expressed genes between groups, then performed pathway enrichment analysis and used the resulting enriched terms as pathway-level features for downstream classification. We have compared the functional categories identified by SDAN versus those identified by DE + enrichment methods. In addition, we also compared classification accuracy. Across five benchmark settings, SDAN outperformed the classical DE + enrichment workflow in four settings and remained competitive in the fifth setting. Details and results are provided in Supplementary Section 5.

In addition, we have also clarified that SDAN focuses on the gene sets that have different contributions to the two classes of cells. In fact, if some gene sets have equal contributions to the two classes of cells, they will not be reported by SDAN. We have added the following clarifications in the Discussion Section of the revised manuscript (on page 9).

> In addition, as a supervised gene program learning method, SDAN focuses on gene programs that are associated with the phenotype of interest and will not detect gene programs that have similar activities across different values of the phenotype.

4. An interesting aspect of SDAN is that the learned mapping matrix S is cell independent. In principle, this suggests that the model could be applied to other datasets that share the same gene measurements. Beyond evaluating performance through a train–test split within the same dataset, it would be valuable to assess the generalizability of SDAN on more biologically heterogeneous datasets, such as across independent studies. Such analysis would provide stronger evidence of the robustness and transferability of the learned representation.

We thank the reviewer for raising the important point regarding cross-dataset generalizability. We agree that the cell-independent loading matrix S learned by SDAN naturally allows the learned representation to be transferred to other datasets. In fact, we have already evaluated this capability in the cancer immunotherapy analysis, where the model was trained and tested on independent studies. Specifically, SDAN was trained on the dataset from Sade-Feldman et al. (2018) and evaluated on an independent dataset from Yost et al. (2019). SDAN successfully transferred the learned gene-set representation to the external dataset and produced meaningful predictions.

We have discussed this scenario in the "Evaluation and generalization" sub-section of the Methods Section (on page 11). In this sub-section, we describe how SDAN can be applied when the training and testing datasets differ in both cells and gene lists. When the genes used by the training/testing data are different, SDAN identifies the common genes between the training and testing datasets and applies the corresponding submatrix of the learned loading matrix S to project the new data into the learned latent gene-set space before prediction. This design ensures that the learned representation can be applied across datasets with partially overlapping gene measurements.

To make this point clear to readers, we have revised the manuscript to explicitly highlight that

the cancer immunotherapy experiment serves as a cross-study validation, demonstrating the robustness and transferability of SDAN across independent datasets. We have added the following text into the Section of "Gene expression of CD8+ T cells can predict cancer patients' response to immunotherapy" (on page 7).

> Notably, this analysis also evaluates the cross-study generalizability of SDAN. The model was trained on the dataset from Sade-Feldman et al. (2018) and tested on the independent dataset from Yost et al. (2019), which was generated in a separate study with different patients and cells. This cross-study evaluation provides a direct assessment of the robustness and transferability of SDAN when applying the learned gene-set representation to external datasets.

We have also added the following text to the "Evaluation and generalization" subsection of the Method Section (on page 11).

> A key advantage of SDAN is that the learned loading matrix S, which maps genes to gene programs, is independent of individual cells and can therefore be reused across datasets. When applying the model to a new dataset, we align the gene lists between the training and test data and project the test data into the learned latent space using the corresponding submatrix of S. This design allows SDAN to generalize to datasets containing different cells and potentially different, but overlapping, gene sets.

5. In lines 171-173, the manuscript states that "Combining pTau and amyloid beta measurement may give a better characterization of the dementia-i donors (Fig 3(H)), though a larger sample size is needed to confirm this conclusion." Does the current modeling framework allow for the integration of two or more cell types simultaneously, and if so, how would it be extended to accommodate such multi-cell-type inputs?

Thank you for this important comment. In the current manuscript, SDAN is applied to one cell type at a time. In the dementia analysis, astrocytes and microglia/PVM were modeled separately, and their donor-level prediction scores were subsequently compared in Fig. 3(E); they were not used as simultaneous inputs to a single SDAN model. This is consistent with the current formulation of SDAN, in which the model is defined for a single expression matrix X, a single adjacency matrix A, and a single loading matrix S, with donor-level prediction obtained by averaging cell-level scores within each individual.

That said, the SDAN framework can be extended in at least two principled ways to accommodate multiple cell types.

First, one can fit a separate SDAN model for each cell type and then combine the resulting donor-level prediction scores in a downstream meta-classifier. In fact, Fig. 3(E) already suggests that combining donor-level information across cell types may improve classification or help identify clinically relevant patient subgroups, even though such an integrative classifier was not formally implemented in the current study.

Second, SDAN can be generalized to a joint multi-branch architecture that models multiple cell types simultaneously. In this extension, each cell type k would have its own input expression matrix $X^{(k)}$, gene graph $A^{(k)}$, and loading matrix $S^{(k)}$. Each branch would learn cell-type-specific latent gene programs, for example through $Z^{(k)} = X^{(k)}S^{(k)}$. The resulting cell-level latent representations

or prediction scores could then be aggregated within each donor and cell type and passed to a shared donor-level classifier. The full model would be trained jointly by optimizing the combined loss across all branches, thereby learning a separate loading matrix $S^{(k)}$ for each cell type while integrating complementary signals at the donor level.

Under either extension, donor-level neuropathology variables such as pTau and amyloid-β could also be incorporated as additional donor-level covariates in the final classification step. In summary, the current implementation does not jointly model multiple cell types within a single SDAN fit, but the underlying framework is readily extensible to this setting.

To address this point, we have now added the following text to the "Extensions" sub-section of Methods Section (last paragraph of "Extensions" sub-section, on page 12):

> In the current implementation, SDAN is applied to one cell type at a time. When the scientific objective is to integrate information across multiple cell types, one natural extension is to fit separate SDAN models for each cell type, aggregate cell-level prediction scores within each donor and cell type, and then combine these donor-level scores using a downstream meta-classifier. Alternatively, SDAN can be extended to a multi-branch architecture with one cell-type-specific branch for each cell type, followed by a shared donor-level integration layer that is optimized jointly across branches. These extensions would preserve the interpretability of cell-type-specific gene programs while enabling joint prediction based on complementary signals from multiple cell types.

Minor comments

1. The gene-gene network is constructed from PPI. It is unclear how this information is mapped or utilized at the gene level in the proposed framework. Clarification on this point would be helpful.

Thank you for the comment. The original interaction data come from PPI databases, but in our framework these interactions are mapped to the gene level: each protein is linked to its corresponding gene, and interactions are converted into gene-gene edges. If multiple protein isoforms map to the same gene, they are collapsed into one gene node.

To clarify this step, we have now added the following text to the "Implementation of SDAN" sub-section of Methods Section:

> The gene–gene interaction graph was constructed using protein–protein interaction annotations from the BioGRID database, specifically the file `"BIOGRID-ORGANISM-Homo_sapiens-4.4.204.tab3.txt.gz"`. Genes were matched by their official gene symbols in BioGRID. Although BioGRID records interactions based on proteins, each protein entry is linked to a corresponding gene symbol. In cases where multiple protein isoforms corresponded to the same gene, these isoforms were collapsed into a single gene-level node. An edge was placed between two genes if an interaction between any of their encoded proteins was recorded in the database. Thus, the final graph is a gene-level interaction network derived from protein-level evidence.

References

[1] Delaram Pouyabahar, Tallulah Andrews, and Gary D Bader. Interpretable single-cell factor decomposition using sciRED. *Nature communications*, 16(1):1878, 2025.

[2] Shuo Feng, Daniel J Phillips, Thomas White, Homesh Sayal, Parvinder K Aley, Sagida Bibi, Christina Dold, Michelle Fuskova, Sarah C Gilbert, Ian Hirsch, et al. Correlates of protection against symptomatic and asymptomatic SARS-CoV-2 infection. *Nature medicine*, 27(11):2032–2040, 2021.

[3] David S Khoury, Deborah Cromer, Arnold Reynaldi, Timothy E Schlub, Adam K Wheatley, Jennifer A Juno, Kanta Subbarao, Stephen J Kent, James A Triccas, and Miles P Davenport. Neutralizing antibody levels are highly predictive of immune protection from symptomatic SARS-CoV-2 infection. *Nature medicine*, 27(7):1205–1211, 2021.

[4] Peter B Gilbert, David C Montefiori, Adrian B McDermott, Youyi Fong, David Benkeser, Weiping Deng, Honghong Zhou, Christopher R Houchens, Karen Martins, Lakshmi Jayashankar, et al. Immune correlates analysis of the mRNA-1273 COVID-19 vaccine efficacy clinical trial. *Science*, 375(6576):43–50, 2022. PMCID: PMC9017870.

[5] F Javier Ibarrondo, Jennifer A Fulcher, David Goodman-Meza, Julie Elliott, Christian Hofmann, Mary A Hausner, Kathie G Ferbas, Nicole H Tobin, Grace M Aldrovandi, and Otto O Yang. Rapid decay of anti–sars-cov-2 antibodies in persons with mild covid-19. *New England Journal of Medicine*, 383(11):1085–1087, 2020.

[6] Markus Hoffmann, Nadine Krüger, Sebastian Schulz, Anne Cossmann, Cheila Rocha, Amy Kempf, Inga Nehlmeier, Luise Graichen, Anna-Sophie Moldenhauer, Martin S Winkler, et al. The omicron variant is highly resistant against antibody-mediated neutralization–implications for control of the COVID-19 pandemic. *Cell*, 185(3):447–456.e11, 2022.

[7] Alessandro Sette and Shane Crotty. Adaptive immunity to SARS-CoV-2 and COVID-19. *Cell*, 184(4):861–880, 2021.

[8] Carolyn Rydyznski Moderbacher, Sydney I Ramirez, Jennifer M Dan, Alba Grifoni, Kathryn M Hastie, Daniela Weiskopf, Simon Belanger, Robert K Abbott, Christina Kim, Jinyong Choi, et al. Antigen-specific adaptive immunity to sars-cov-2 in acute covid-19 and associations with age and disease severity. *Cell*, 183(4):996–1012, 2020.

[9] Anthony T Tan, Martin Linster, Chee Wah Tan, Nina Le Bert, Wan Ni Chia, Kamini Kunasegaran, Yan Zhuang, Christine YL Tham, Adeline Chia, Gavin JD Smith, et al. Early induction of functional SARS-CoV-2-specific T cells associates with rapid viral clearance and mild disease in COVID-19 patients. *Cell reports*, 34(6):108728, 2021.

[10] Erin M Bange, Nicholas A Han, Paul Wileyto, Justin Y Kim, Sigrid Gouma, James Robinson, Allison R Greenplate, Madeline A Hwee, Florence Porterfield, Olutosin Owoyemi, et al. CD8 + T cells contribute to survival in patients with COVID-19 and hematologic cancer. *Nature medicine*, 27(7):1280–1289, 2021.

[11] Valerie Oberhardt, Hendrik Luxenburger, Janine Kemming, Isabel Schulien, Kevin Ciminski, Sebastian Giese, Benedikt Csernalabics, Julia Lang-Meli, Iga Janowska, Julian Staniek, et al. Rapid and stable mobilization of CD8+ T cells by SARS-CoV-2 mRNA vaccine. *Nature*,

597(7875):268–273, 2021.

[12] Alison Tarke, Camila H Coelho, Zeli Zhang, Jennifer M Dan, Esther Dawen Yu, Nils Methot, Nathaniel I Bloom, Benjamin Goodwin, Elizabeth Phillips, Simon Mallal, et al. SARS-CoV-2 vaccination induces immunological T cell memory able to cross-recognize variants from alpha to omicron. *Cell*, 185(5):847–859, 2022.

[13] Paul Moss. The T cell immune response against SARS-CoV-2. *Nature immunology*, 23(2):186–193, 2022.

[14] Jonas Schulte-Schrepping, Nico Reusch, Daniela Paclik, Kevin Baßler, Stephan Schlickeiser, Bowen Zhang, Benjamin Krämer, Tobias Krammer, Sophia Brumhard, Lorenzo Bonaguro, et al. Severe covid-19 is marked by a dysregulated myeloid cell compartment. *Cell*, 182(6):1419–1440, 2020.

[15] Colin L Masters, Randall Bateman, Kaj Blennow, Christopher C Rowe, Reisa A Sperling, and Jeffrey L Cummings. Alzheimer's disease. *Nature reviews disease primers*, 1(1):1–18, 2015.

[16] Michael T Heneka, Wiesje M Van der Flier, Frank Jessen, Jeroen Hoozemanns, Dietmar Rudolf Thal, Delphine Boche, Frederic Brosseron, Charlotte Teunissen, Henrik Zetterberg, Andreas H Jacobs, et al. Neuroinflammation in alzheimer disease. *Nature Reviews Immunology*, 25(5):321–352, 2025.

[17] Soyon Hong, Victoria F Beja-Glasser, Bianca M Nfonoyim, Arnaud Frouin, Shaomin Li, Saranya Ramakrishnan, Katherine M Merry, Qiaoqiao Shi, Arnon Rosenthal, Ben A Barres, et al. Complement and microglia mediate early synapse loss in alzheimer mouse models. *Science*, 352(6286):712–716, 2016.

[18] Naomi Habib, Cristin McCabe, Sedi Medina, Miriam Varshavsky, Daniel Kitsberg, Raz Dvir-Szternfeld, Gilad Green, Danielle Dionne, Lan Nguyen, Jamie L Marshall, et al. Disease-associated astrocytes in alzheimer's disease and aging. *Nature neuroscience*, 23(6):701–706, 2020.

[19] Shane A Liddelow, Kevin A Guttenplan, Laura E Clarke, Frederick C Bennett, Christopher J Bohlen, Lucas Schirmer, Mariko L Bennett, Alexandra E Münch, Won-Suk Chung, Todd C Peterson, et al. Neurotoxic reactive astrocytes are induced by activated microglia. *Nature*, 541(7638):481–487, 2017.

[20] Patrick L McGeer, Shigeru Itagaki, Hisao Tago, and Edith G McGeer. Reactive microglia in patients with senile dementia of the alzheimer type are positive for the histocompatibility glycoprotein hla-dr. *Neuroscience letters*, 79(1-2):195–200, 1987.

[21] Douglas P Wightman, Iris E Jansen, Jeanne E Savage, Alexey A Shadrin, Shahram Bahrami, Dominic Holland, Arvid Rongve, Sigrid Børte, Bendik S Winsvold, Ole Kristian Drange, et al. A genome-wide association study with 1,126,563 individuals identifies new risk loci for alzheimer's disease. *Nature genetics*, 53(9):1276–1282, 2021.
